# Restructuring‐Regulated Bismuth Catalyst Promotes Electrochemical CO_2_ Reduction to Formic Acid in Acidic Electrolyte

**DOI:** 10.1002/smsc.202500565

**Published:** 2026-01-19

**Authors:** Ganwen Chen, Chun Liu, Jie Chen, Yukun Xiao, Yumin Da, Meng Wang, Chenrui Ji, Jie He, Rongjie Xu, Lei Fan, Zhangliu Tian, Wei Chen

**Affiliations:** ^1^ Joint School of National University of Singapore and Tianjin University International Campus of Tianjin University Binhai New City Fuzhou 350207 P. R. China; ^2^ Department of Chemistry National University of Singapore 3 Science Drive 3 Singapore 117543 Singapore; ^3^ State Key Laboratory of Metal Organic Chemistry Shanghai Institute of Organic Chemistry Chinese Academy of Sciences Shanghai 200032 P. R. China; ^4^ Northwest Institute for Non‐ferrous Metal Research Xi'an 710016 P. R. China; ^5^ Advanced Institute for Materials Research (WPI‐AIMR) Tohoku University Sendai 980‐8577 Japan; ^6^ CAS Key Laboratory of Materials for Energy Conversion Shanghai Institute of Ceramics Chinese Academy of Sciences Shanghai 200050 P. R. China; ^7^ Department of Physics National University of Singapore 2 Science Drive 3 Singapore 117542 Singapore

**Keywords:** carbon dioxide reduction, electrocatalysis, formic acid, structure restructuring

## Abstract

Electrochemical CO_2_ reduction (eCO_2_R) in acidic electrolytes is appealing due to its high CO_2_ utilization efficiency. For this reaction, bismuth (Bi)‐based catalysts have drawn considerable attention for their potential in producing formate/formic acid. However, the presynthesized materials for Bi‐based catalysts often undergo restructuring during electrocatalysis, resulting in altered electrochemical performance. Furthermore, the mechanisms underlying the restructuring of Bi‐based catalysts in acidic environments have not yet been clearly elucidated. Herein, distinct restructuring mechanisms are revealed in structurally different Bi‐based compounds, such as Bi_9_O_7.5_S_6_ and Bi_2_O_2_S. Among them, the Bi_9_O_7.5_S_6_ precatalyst exhibits high selectivity and activity for formic acid production, attributed to its unique structure, featuring stacking of [Bi_2_O_2_]^2+^ and [BiS_2_]^−^ layers. In contrast, the conventional Bi_2_O_2_S catalyst, characterized by alternating [Bi_2_O_2_]^2+^ layers with S^2−^ ions, delivers inferior eCO_2_R performances. Quasi‐in situ X‐ray diffraction and in situ Raman spectra results reveal that metal elements situated between two [Bi_2_O_2_]^2+^ layers can resist decomposition and prevent the over‐reduction of catalysts, leading to the restructuring in Bi/Bi_2_O_2_CO_3_ composite material with active Bi‐Bi_2_O_2_CO_3_ interface for formic acid production. As a result, the Bi_9_O_7.5_S_6_ precatalyst achieves a high Faraday efficiency above 95% at 100 mA cm^−2^ and remarkable stability of 117 h in a flow cell.

## Graphical abstract

Two Bi‐based oxysulfides, Bi_9_O_7.5_S_6_ and Bi_2_O_2_S, undergo distinct restructuring under acidic eCO_2_R. Bi within the [BiS_2_]^−^ layers of Bi_9_O_7.5_S_6_ guides the formation of an active Bi/Bi_2_O_2_CO_3_ interface, whereas Bi_2_O_2_S mainly reduces to Bi metal, giving lower activity for formic acid production.

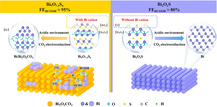

## 1 Introduction

The utilization of carbon dioxide (CO_2_) as a feedstock offers a promising route toward carbon neutralization by enabling its electrocatalytic reduction into more valuable carbon‐based compounds.^[^ ^1^, ^2^, ^3^ ^]^ Depending on the type of metal catalysts and factors such as surface reconstruction, carbon deposition, and grain‐boundary engineering, CO_2_ can be converted into a variety of products, including C_1_ products (such as CO, formic acid, and methane) and multicarbon (C_2+_) products (such as ethylene, ethanol, and propanol).^[^ ^4^, ^5^ ^]^ Within the realm of the chemical industry, the synthesis of formic acid from carbon dioxide holds particular significance due to its high market value.^[^ ^6^ ^]^ Most prior studies have focused on inhibiting the hydrogen evolution reaction (HER) to enhance the selectivity of electrocatalytic CO_2_ reduction (eCO_2_R) in alkaline or near‐neutral solutions.^[^ ^7^ ^]^ However, these conditions pose practical challenges.^[^ ^8^, ^9^ ^]^ The input CO_2_ will react with OH^−^ to produce carbonate or bicarbonate, commonly referred to as the carbonation problem. In alkaline electrolytes, the spontaneous reaction between alkali and CO_2_ can cause a substantial carbon loss of up to 50%.^[^ ^10^ ^]^ The formed carbonate/bicarbonate precipitate may block the porous channels for CO_2_ transport in gas diffusion electrodes (GDEs).^[^ ^9^, ^11^ ^]^ Additionally, the production of formate requires subsequent acidification and separation processes to yield pure formic acid products.^[^ ^12^ ^]^ In contrast, acidic electrolytes enable the direct production of formic acid without requiring additional acidification and alleviate the issue of carbonation formation.^[^ ^13^, ^14^, ^15^ ^]^ However, in an extremely acidic environment, the rapid HER leads to a decrease in the Faraday efficiency (FE) for converting CO_2_ into formic acid.^[^ ^16^ ^]^ Therefore, designing a robust catalyst with high efficiency for formic acid production and low HER activity under acidic conditions is crucial.

To date, metal‐based catalysts, including Bi, Sn, In, and their compounds, have demonstrated significant advantages in the electrocatalytic conversion of CO_2_ to formate. Among them, Bi‐based electrocatalysts have received the widest attention due to their low toxicity, low cost, and high selectivity for formate.^[^ ^17^ ^]^ It has been noted that most Bi‐based electrocatalysts undergo surface restructuring into metallic Bi or Bi_2_O_2_CO_3_ in neutral and alkaline environments.^[^ ^18^, ^19^, ^20^ ^]^ However, in an acidic environment, the component of the catalysts after electrocatalysis remains uncertain, posing challenges for a comprehensive understanding of the eCO_2_R activity under acidic conditions.^[^ ^21^, ^22^, ^23^ ^]^

Sulfur‐containing Bi‐based catalysts have exhibited superior performance for formate production.^[^ ^24^, ^25^, ^26^ ^]^ In these systems, S^2−^ anions are leached during eCO_2_R, thereby modifying the catalyst/electrolyte interface and accelerating restructuring toward Bi and Bi_2_O_2_CO_3_ phases.^[^ ^27^, ^28^ ^]^ Bi_2_O_2_S is a typical Bi‐based oxysulfide material, characterized by alternating layers of [Bi_2_O_2_]^2+^ with S^2−^ ions. Sulfur ions within this structure can be easily removed via reduction during the eCO_2_R process, leading to surface structural evolution within Bi_2_O_2_S.^[^ ^28^ ^]^ A novel Bi‐based oxysulfide material, Bi_9_O_7.5_S_6_, also features a layered structural framework similar to that of Bi_2_O_2_S. However, the key difference is that the [Bi_2_O_2_]^2+^ layers in Bi_9_O_7.5_S_6_ are interspersed with [BiS_2_]^−^ layers containing trivalent bismuth elements. This structural distinction can lead to different surface structure evolution during eCO_2_R, ultimately resulting in varying surface catalytic activity and overall performance.

**Figure 1:**
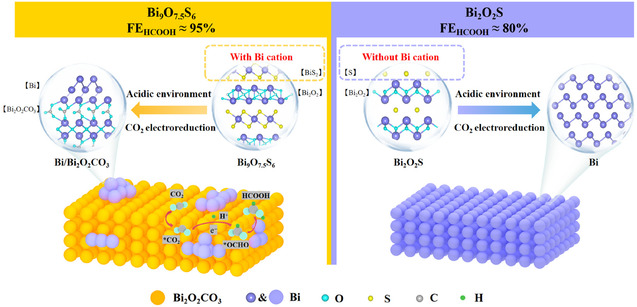
Schematic illustration depicting the structures of Bi_9_O_7.5_S_6_ and Bi_2_O_2_S catalysts and their respective restructuring processes and final structures.

Herein, two distinct restructuring mechanisms in acid environments are revealed by the two structurally different Bi‐based oxysulfur compounds, Bi_9_O_7.5_S_6_ and Bi_2_O_2_S. The novel Bi_9_O_7.5_S_6_ compound features alternating stacking of [Bi_2_O_2_]^2+^ and [BiS_2_]^−^ layers with trivalent bismuth elements in each layer can prevent the reduction of [Bi_2_O_2_]^2+^ and promote its restructuring into Bi/Bi_2_O_2_CO_3_ composite material (**Figure** **1**). The conventional Bi_2_O_2_S compound, however, tends to be restructured into metal Bi due to its original structure of alternating [Bi_2_O_2_]^2+^ layers with S^2−^ ions. Our results demonstrate that the restructured Bi_9_O_7.5_S_6_ (Bi_9_O_7.5_S_6_^R^) possesses higher catalytic activity for formic acid production than the restructured Bi_2_O_2_S (Bi_2_O_2_S^R^). The Bi_9_O_7.5_S_6_^R^ catalyst achieves a high formic acid FE (FE_HCOOH_) of above 95% at 100 mA cm^−2^ with remarkable stability achieving 117 h in a flow cell, while the Bi_2_O_2_S^R^ catalyst delivers a lower FE_HCOOH_ of about 80%. In situ spectroscopy studies and isotope‐labeling experiment confirm that *OCHO is the key intermediate, and its protonation to formic acid is the rate‐determining step (RDS). Theoretical calculations demonstrate that the active interface of Bi_9_O_7.5_S_6_^R^ favored the conversion of *OCHO to HCOOH.

## 2 Results and Discussion

### 2.1 Structural Characterizations of Bi_9_O_7.5_S_6_ and Bi_2_O_2_S Nanosheets

**Figure 2:**
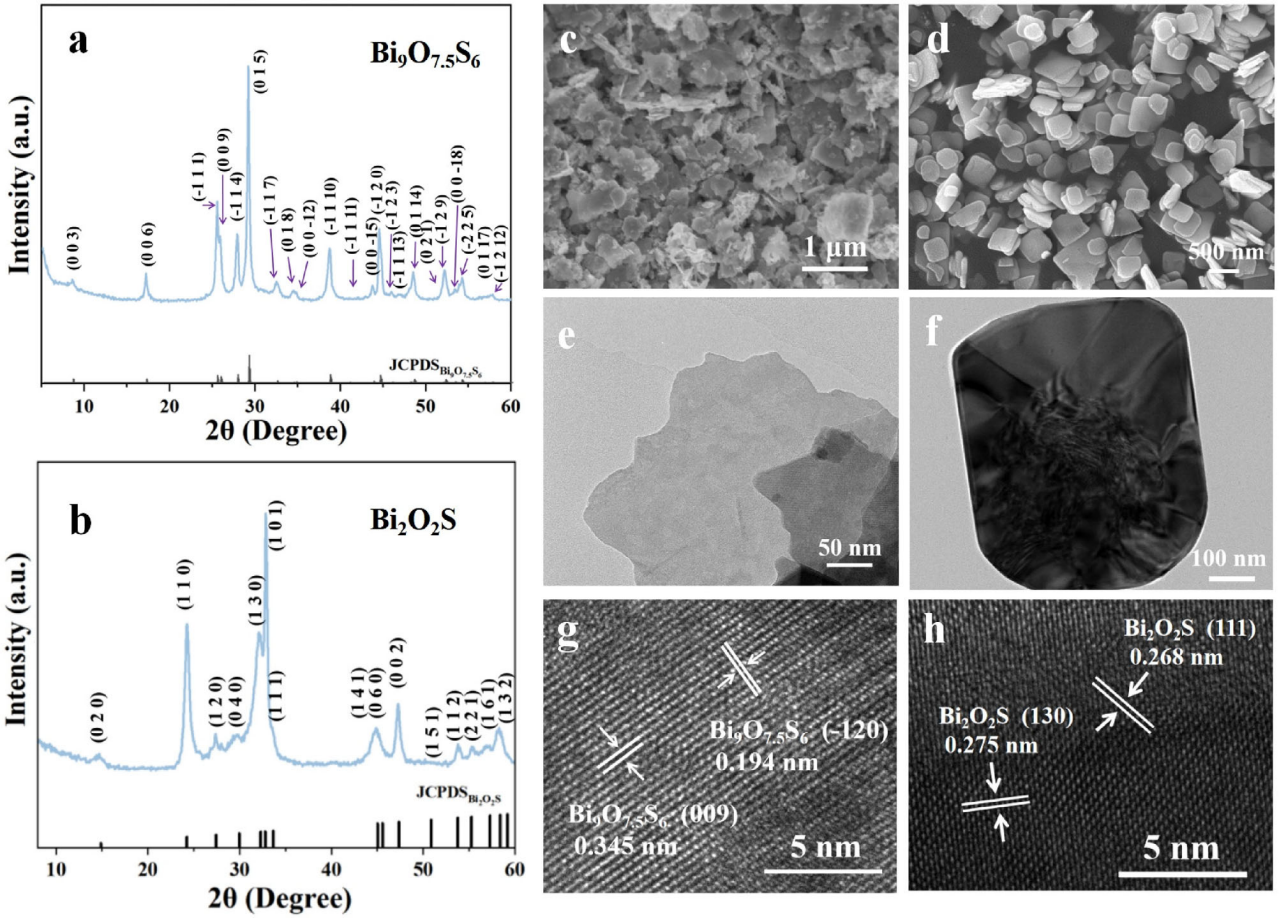
XRD patterns of the a) Bi_9_O_7.5_S_6_ and b) Bi_2_O_2_S NSs. SEM images of the c) Bi_9_O_7.5_S_6_ and d) Bi_2_O_2_S NSs. TEM images of the single crystal of e) Bi_9_O_7.5_S_6_ and f) Bi_2_O_2_S. HRTEM images of the g) Bi_9_O_7.5_S_6_ and h) Bi_2_O_2_S.

The Bi_9_O_7.5_S_6_ and Bi_2_O_2_S nanosheets (NSs) were synthesized via one‐step solvothermal methods under different conditions. The structure and phase purity of the resulting samples were confirmed by X‐ray diffraction (XRD) patterns. As shown in **Figure** **2**a, the powder XRD pattern of Bi_9_O_7.5_S can be well indexed to the reported trigonal Bi_9_O_7.5_S_6_ and the patterns of Bi_2_O_2_S (Figure 2b) align well with the standard PDF card of orthorhombic Bi_2_O_2_S (PDF#34‐1493), confirming the successful synthesis of Bi_9_O_7.5_S_6_ and Bi_2_O_2_S NSs.^[^ ^29^, ^30^ ^]^ Field‐emission scanning electron microscopy (FE‐SEM) images demonstrate that Bi_9_O_7.5_S_6_ consists of irregularly shaped nanosheets with particle sizes ranging from 100 to 1000 nm (Figure 2c), while Bi_2_O_2_S consists of square nanosheets with a side length varying ranging from 200 to 500 nm (Figure 2d). The transmission electron microscopy (TEM) images (Figure 2e,f) further confirm the sheet‐like morphology of the obtained Bi_9_O_7.5_S_6_ and Bi_2_O_2_S. High‐resolution transmission electron microscopy (HRTEM) images reveal the lattice fringes of 0.345 and 0.194 nm in Bi_9_O_7.5_S_6_ (Figure 2g), corresponding to the (009) and (−120) crystal planes. In Bi_2_O_2_S, lattice fringes of 0.268 and 0.275 nm (Figure 2h) correspond to the (111) and (130) crystal planes. The energy dispersive X‐ray spectroscopy (EDX) elemental mapping images of Bi_9_O_7.5_S_6_ and Bi_2_O_2_S confirm a uniform distribution of O, S, and Bi elements throughout the whole nanosheet (Figure S1, Supporting Information).

**Figure 3:**
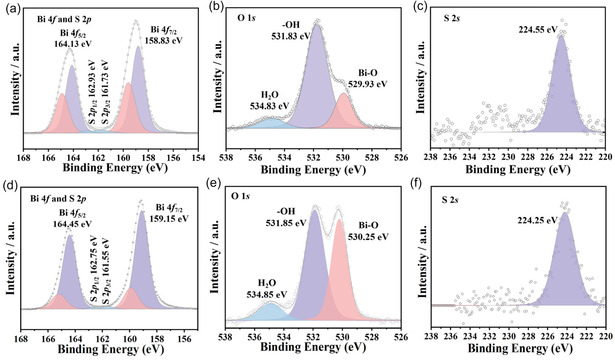
XPS spectra of a) Bi 4*f* and S 2*p*, b) O 1*s*, and c) S 2*s* of the Bi_9_O_7.5_S_6_ catalyst and d) Bi 4*f* and S 2*p*, e) O 1*s*, and f) S 2*s* of the Bi_2_O_2_S catalyst before eCO_2_R.

We then used X‐ray photoelectron spectroscopy (XPS) to investigate the electronic structure of Bi_9_O_7.5_S_6_ and Bi_2_O_2_S. As shown in **Figure** **3**a, the dominant peaks in the Bi 4*f* spectra can be deconvoluted into four subpeaks at 158.83 (Bi—S), 159.63 (Bi—O), 164.13 (Bi—S), and 164.93 (Bi—O) eV, respectively.^[^ ^30^ ^]^ The O 1*s* XPS spectrum of pristine Bi_9_O_7.5_S_6_ (Figure 3b) can be split into three deconvolution peaks at ≈529.93, 531.83, and 534.83 eV, which belong to Bi—O, Bi‐OH, and surface‐adsorbed H_2_O, respectively.^[^ ^31^, ^32^ ^]^ Since the Bi 4*f* orbital overlaps with the S 2*p* orbital, S 2*s* spectrum was tested, which can be fitted into a peak at 224.55 eV (Figure 3c).^[^ ^33^ ^]^ In comparison, the Bi_2_O_2_S NSs show Bi 4*f* peaks at 159.15 (Bi—S), 159.85 (Bi—O), 164.45 (Bi—S), and 165.25 (Bi—O) eV, respectively (Figure 3d); O 1*s* peaks at 530.25 (Bi—O), 531.85 (Bi—OH), and 534.85 (surface‐adsorbed H_2_O) eV (Figure 3e); and S 2*s* peaks at 224.25 eV (Figure 3f). The Bi_9_O_7.5_S_6_ NSs exhibit obviously lower binding energy for Bi 4*f* peaks than the Bi_2_O_2_S NSs, probably due to the formed Bi—S covalent bond in the [BiS_2_]^−^ layers.

### 2.2 Electrochemical Performances

**Figure 4:**
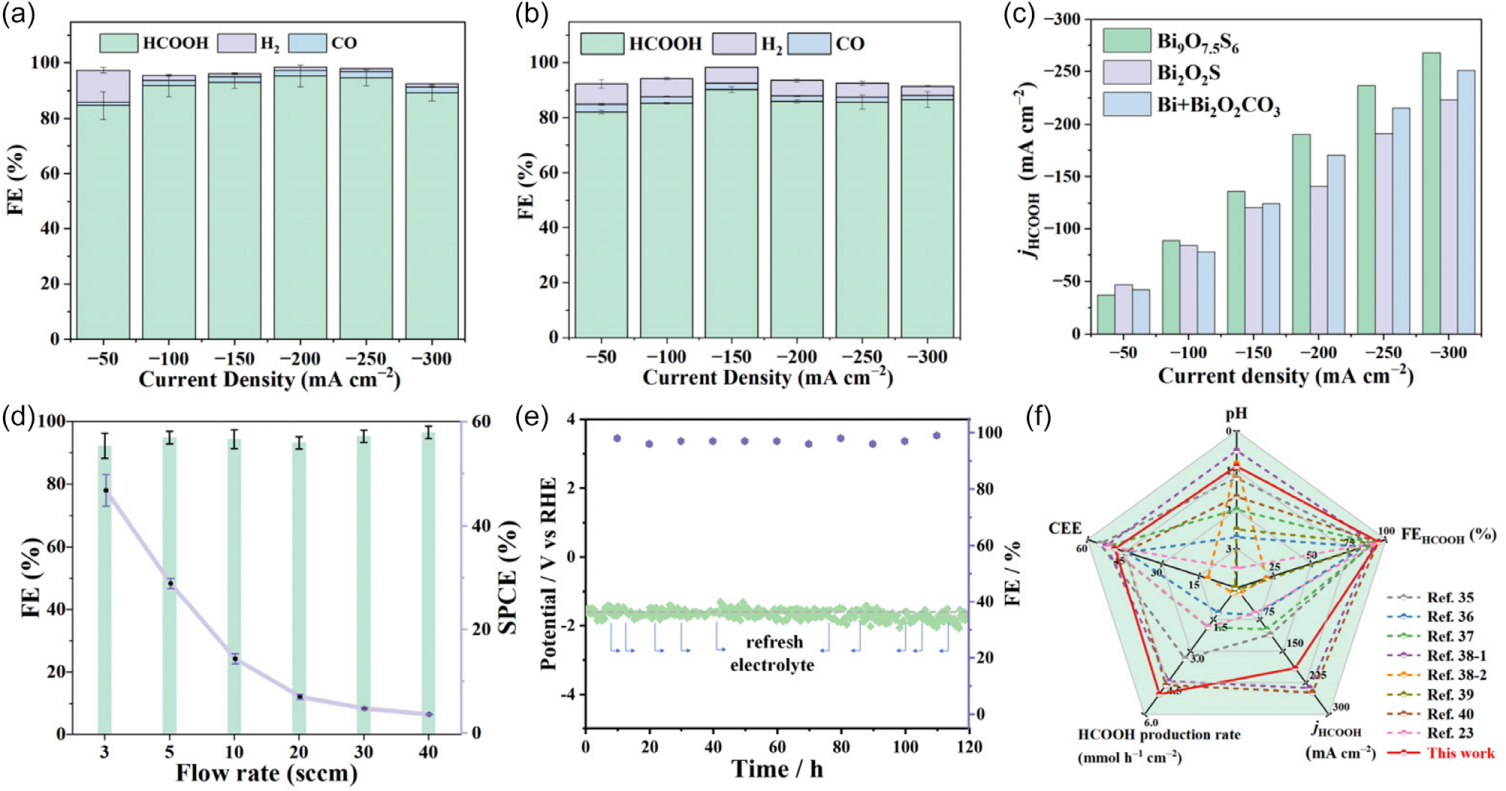
FEs of the a) Bi_9_O_7.5_S_6_ and b) Bi_2_O_2_S catalysts for eCO_2_R to produce HCOOH under strongly acidic conditions (0.05 m H_2_SO_4_ with 0.5 m KCl electrolyte) over a wide current density window in flow cell. c) HCOOH partial current densities of the Bi_9_O_7.5_S_6_, Bi_2_O_2_S and Bi_2_O_2_CO_3_ catalysts in 0.05 m H_2_SO_4_ with 0.5 m KCl electrolyte in flow cell. d) FEs and single‐pass carbon conversion efficiency of the Bi_9_O_7.5_S_6_ catalysts in different CO_2_ gas flow rates in flow cell. e) Electrochemical stability of the Bi_9_O_7.5_S_6_ catalyst at a current density of 100 mA cm^−2^. f) Comparison of cathodic energy efficiency (CEE), pH, HCOOH production rate, FE of HCOOH, and HCOOH partial current density in a flow cell with those of reported Bi‐based catalysts. All data with error bars are based on three independent replicates.

The electrochemical performance of different catalysts was tested in a flow‐type reactor. Linear sweep voltammetry (LSV) curves of the pristine Bi, Bi_2_O_2_CO_3_, and Bi_9_O_7.5_S_6_ were conducted to evaluate their eCO_2_R performance over a potential range of −0.4 to −1.4 V_RHE_, where a three‐electrode flow cell reactor was employed with 0.05 m H_2_SO_4_ as the anode electrolyte and 0.05 m H_2_SO_4_ with 0.5 m KCl as the cathode electrolyte, respectively. As shown in Figure S2, Supporting Information, the Bi_9_O_7.5_S_6_ electrode shows higher cathodic current densities for eCO_2_R in CO_2_ than in Ar. The electrochemical activity of all materials in a CO_2_ atmosphere is improved, with the Bi_9_O_7.5_S_6_ material showing the most notable enhancement. However, the performance of the intrinsic state materials is inferior to that of commercial Bi powder and Bi_2_O_2_CO_3_. To investigate the actual active sites of the precatalysts, all tested catalysts were first pretreated and activated for 30 min at 50 mA cm^−2^ before performing the eCO_2_R performance tests. Since alkaline metal ions play a crucial role in acidic eCO_2_R, we first investigated the influence of K^+^ concentration on the electrocatalytic performance of the Bi_9_O_7.5_S_6_ catalyst. As shown in Figure S3a, Supporting Information, the Bi_9_O_7.5_S_6_ catalyst yields a maximum FE_HCOOH_ of 80% in the electrolyte with 0.1 m K^+^. When the K^+^ concentration was increased to 0.5 m, the Bi_9_O_7.5_S_6_ catalyst shows significant enhancement in the maximum FE_HCOOH_, exceeding 95% (**Figure** **4**a). Further increasing the K^+^ concentration to 1 m did not affect the eCO_2_R performance (Figure S3b, Supporting Information). Consequently, an electrolyte composition of 0.5 m KCl and 0.05 M H_2_SO_4_ was used for subsequent eCO_2_R investigations.

The Bi_9_O_7.5_S_6_ catalyst exhibits outstanding formic acid selectivity over a wide current density window. As shown in Figure 4a,b, the Bi_9_O_7.5_S_6_ catalyst shows obviously superior selectivity for producing formic acid compared to the Bi_2_O_2_S catalyst at all the same applied current densities resulting in higher HCOOH partial current densities (*J*_HCOOH_) of Bi_9_O_7.5_S_6_ than those of Bi_2_O_2_S (Figure 4c). Impressively, the Bi_9_O_7.5_S_6_ catalyst achieves a high FE_HCOOH_ of over 95% at 200 mA cm^−2^ while the Bi_2_O_2_S catalyst produces a lower FE_HCOOH_ of around 80% at the same applied current density. This comparison highlights the superior performance of Bi_9_O_7.5_S_6_ in selectively catalyzing formic acid generation above 100 mA cm^−2^. Furthermore, the maximum calculated *J*_HCOOH_ can reach 270 mA cm^−2^ at −1.75 V_RHE_ (Figure 4c and Figure S3c, Supporting Information), implying its potential for industrial applications. The pH value of the electrolyte doesn't change during the catalytic process (Figure S3c, Supporting Information). Moreover, the Bi_9_O_7.5_S_6_ electrode can maintain outstanding formic acid production activity even in more acidic electrolytes with the pH value around 1 (Figure S3d, Supporting Information), indicating its high intrinsic activity.^[^ ^34^ ^]^

The use of acidic electrolytes can inhibit the spontaneous reaction between hydroxide and CO_2_, providing an opportunity to achieve high single‐pass carbon efficiency (SPCE). As a crucial metric for the commercialization of the eCO_2_R process, SPCE directly affects overall costs but is often overlooked in lab‐scale experiments. As shown in Figure 4d, fine‐tuning the CO_2_ gas flow rate to 3 standard cubic centimeters per minute (sccm) enables the Bi_9_O_7.5_S_6_ catalyst to achieve a high SPCE of ≈78%, suggesting that most CO_2_ has been converted into formic acid. Notably, an SPCE exceeding 50% is considered a prerequisite for the commercial viability of the eCO_2_R process, suggesting that our catalyst holds significant potential for practical applications.

The stability of the catalysts significantly impacts the overall cost of the CO_2_‐to‐product system. The durability of the Bi_9_O_7.5_S_6_ electrocatalyst was tested in a flow cell in 0.05 m H_2_SO_4_ with 0.5 m KCl at 100 mA cm^−2^. As shown in Figure 4e, the Bi_9_O_7.5_S_6_ catalyst shows stable potential and FE_HCOOH_ during the 117 h V‐t test with the potential stabilizing at −1.58 V_RHE_ and the FE_HCOOH_ maintaining at 95.3%. This result reveals the stable activity of the Bi_9_O_7.5_S_6_ catalyst for formic acid production. In conclusion, we achieved a *J*_HCOOH_ as high as 190 mA cm^−2^ at a given potential of −1.68 V_RHE_, with a HCOOH formation rate of 5.02 mmol h^−1^ cm^−2^ in acid electrolyte (Figure 4f). This performance is comparable to previously reported acidic electrolyte environment bismuth‐based catalysts, as shown in Figure 4f and Table S123, Supporting Information.^[^ ^35^, ^36^, ^37^, ^38^, ^39^, ^40^ ^]^

### 2.3 Mechanism of Catalyst Restructuring and CO_2_ Reduction

#### 2.3.1 Structural Evolution of the Bi_9_O_7.5_S_6_ and Bi_2_O_2_S NSs during eCO_2_R

**Figure 5:**
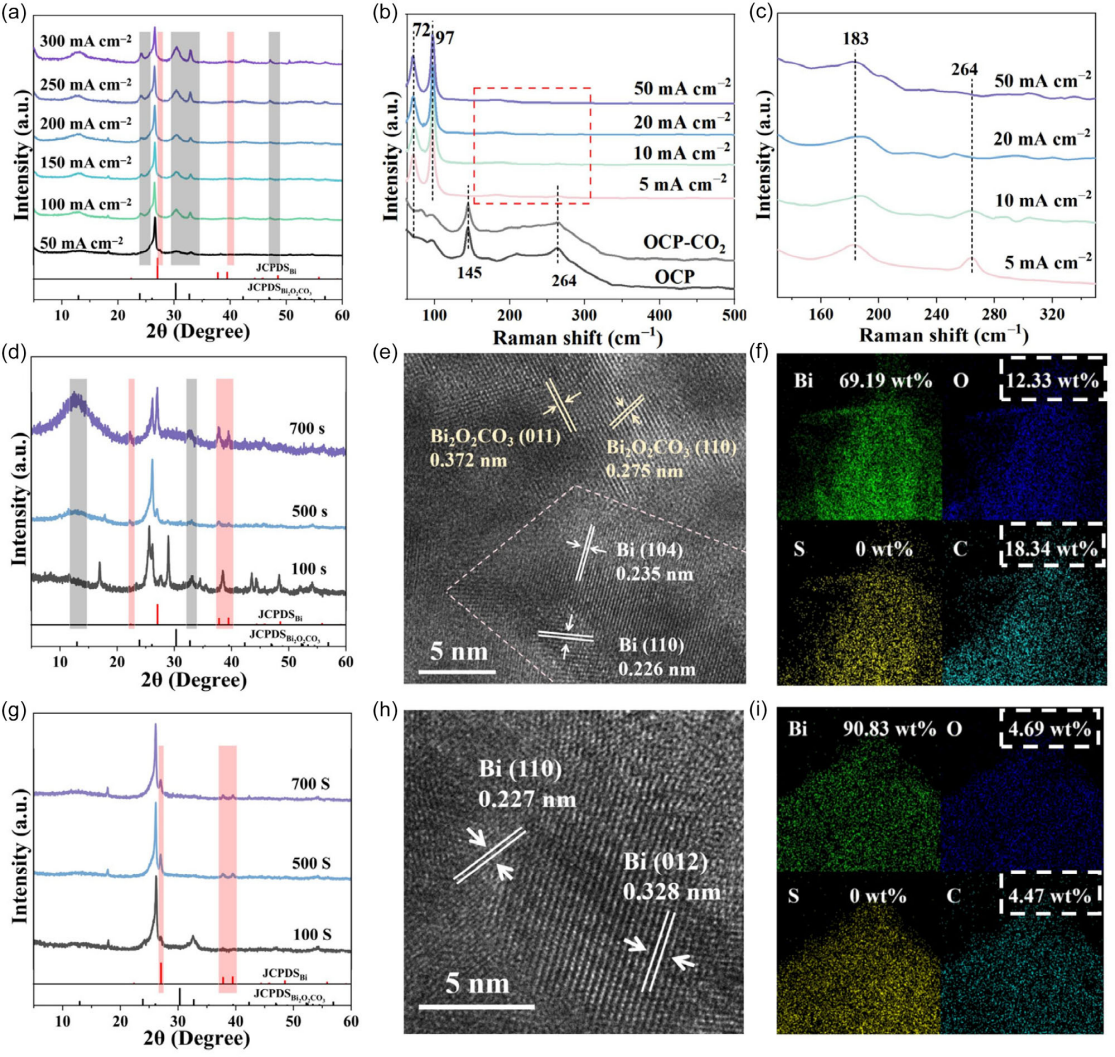
a) Ex situ XRD patterns of the Bi_9_O_7.5_S_6_ electrode under different applied current densities. b,c) In situ Raman spectra of the Bi_9_O_7.5_S_6_ electrode under different applied current densities. d) Quasi‐in situ XRD patterns of the Bi_9_O_7.5_S_6_ electrode at 50 mA cm^−2^ for different times. e) HRTEM image and f) STEM‐EDS elemental mapping of the Bi_9_O_7.5_S_6_ catalyst after eCO_2_R. g) Quasi‐in situ XRD patterns of the Bi_2_O_2_S electrode at 50 mA cm^−2^ for different times. h) HRTEM image and i) STEM‐EDS elemental mapping of the Bi_2_O_2_S catalyst after eCO_2_R. All the quasi‐in situ XRD patterns and in situ Raman spectra were tested in 0.05 m H_2_SO_4_ as the anode electrolyte and 0.05 m H_2_SO_4_ with 0.5 m KCl as the cathode electrolyte. The gray and red bars from all the XRD patterns highlight the peaks of Bi_2_O_2_CO_3_ and Bi, respectively.

Metal oxide or metal sulfide‐based electrocatalysts inevitably undergo surface restructuring at negative potential during eCO_2_R.^[^ ^19^, ^28^, ^41^ ^]^ Despite their similar elemental compositions and atomic structures, the difference in eCO_2_R catalytic efficiency between Bi_9_O_7.5_S_6_ and Bi_2_O_2_S is attributed to their structural differences after restructuring. To investigate the structure evolution of our catalysts, various ex situ and in situ characterization techniques have been employed. Ex situ XRD was first to detect the restructuring of the Bi_9_O_7.5_S_6_ electrode under different applied current densities. As shown in **Figure** **5**a, emerging Bi (PDF#85‐1329) and Bi_2_O_2_CO_3_ (PDF#84‐1752) peaks are observed in all the Bi_9_O_7.5_S_6_ electrodes at different applied current densities. In situ Raman spectra in Figure 5b also demonstrate the structure evolution of the Bi_9_O_7.5_S_6_ catalysts at different applied current densities. Two Raman peaks of the Bi_9_O_7.5_S_6_ electrodes are observed at open‐circuit potential (OCP), with the Raman peaks at 145 and 264 cm^−1^ assigned to the Bi—O bond and Bi—S bond, respectively. Upon applying the current, the in situ Raman spectra of the Bi_9_O_7.5_S_6_ catalysts show the emerging Raman peaks corresponding to Bi—Bi bonds with the *E*_g_ and *A*_1g_ stretching modes at 72 and 97 cm^−1^, as well as Bi—O bond at 183 cm^−1^ with the application of current (Figure 5b,c).^[^ ^42^, ^43^ ^]^ The formation of Bi—Bi and Bi—O bonds upon applying current demonstrates significant structural changes in nanosheets under negative potentials during eCO_2_R. Furthermore, with the increase of applied current densities, the peaks of Bi—S bond at 264 cm^−1^ gradually decrease and even disappear, confirming the breaking of Bi—S bonds during the restructuring. To identify the exact Bi—O intermediates during the restructuring, quasi‐in situ XRD was carried out during eCO_2_R for Bi_9_O_7.5_S_6_ and Bi_2_O_2_S nanosheets at the applied current of 50 mA cm^−2^ for different times (Figure 5d). Quasi‐in situ XRD results obtained at different potentials (Figure S4a, Supporting Information) are consistent with the findings of the time‐dependent experiment. With the extension of electrolysis time, the Bi_9_O_7.5_S_6_ peaks gradually diminish, accompanied by the gradual emergence of Bi and Bi_2_O_2_CO_3_ peaks (Figure 5d).^[^ ^44^ ^]^ This further confirms that Bi_9_O_7.5_S_6_ is gradually transformed into a Bi/Bi_2_O_2_CO_3_ mixture during the eCO_2_R process. These XRD and Raman results reveal that the Bi_9_O_7.5_S_6_ catalyst experiences both reduction and restructuring to Bi/Bi_2_O_2_CO_3_ during the eCO_2_R process.

The morphology of the Bi_9_O_7.5_S_6_^R^ was investigated by SEM, TEM, and HRTEM. As shown in Figure S5a,b, Supporting Information, the restructured Bi_9_O_7.5_S_6_ catalysts demonstrate a nanoflower‐like structure. HRTEM image in Figure 5e shows the lattice constant of 0.278 nm and 0.235 nm, corresponding to the Bi_2_O_2_CO_3_ (110) crystal plane and the Bi (104) crystal plane, respectively. Notably, a large number of Bi/Bi_2_O_2_CO_3_ boundaries are observed in the Bi_9_O_7.5_S_6_ catalyst after the eCO_2_R reaction (Figure S6a, Supporting Information). These results are consistent with the XRD results (Figure 5d and Figure S6c, Supporting Information). Furthermore, the analysis of corresponding EDX elemental mapping of the restructured Bi_9_O_7.5_S_6_ shows the same uniform distribution of O and Bi elements in the whole nanosheet as its precursor except sulfur (Figure 5f), which also aligns with the previous stability test results (Figure S6b, Supporting Information).

For the Bi_2_O_2_S catalyst, only the XRD peaks of metallic Bi are observed with the disappearance of Bi_2_O_2_S peaks, suggesting the formation of Bi in the Bi_2_O_2_S catalyst (Figure 5g). SEM image demonstrates that the Bi_2_O_2_S^R^ electrode consists of nanosheets with average size ranging between 500 nm and 1 μm (Figure S7b, Supporting Information). TEM image (Figure S7c, Supporting Information) of Bi_2_O_2_S exhibits flake structure nanosheets stacked in layers. The spacing of the lattice fringes is 0.324 nm (Figure 5h), corresponding to the distance of the (012) planes of Bi. The final restructured catalyst contains only metallic Bi. EDX mapping of the Bi_2_O_2_S^R^ shows that the most distributed element is Bi (Figure 5i). The XPS spectra of the restructured Bi_9_O_7.5_S_6_ and Bi_2_O_2_S were also tested and shown in Figure S8 and S9, Supporting Information, respectively. The Bi 4*f* binding energy of the restructured Bi_9_O_7.5_S_6_ decreases by 0.10 eV, while the Bi_2_O_2_S^R^ decreases by 0.47 eV. This difference indicates that only a small amount of Bi in the Bi_9_O_7.5_S_6_^R^ is reduced to the zero‐valent state, with a large amount retaining its positive oxidation state (Figure S8a, Supporting Information). In contrast, the Bi of the Bi_2_O_2_S^R^ is almost entirely reduced to the zero‐valent metallic state (Figure S10a, Supporting Information). These results confirmed the formation of Bi/Bi_2_O_2_CO_3_ composite and metallic Bi after the restructuring of Bi_9_O_7.5_S_6_ and Bi_2_O_2_S, respectively. Therefore, in acidic environment, the Bi/Bi_2_O_2_CO_3_ composite is evidenced to enhance the activity of eCO_2_R toward formic acid production, which accounts for the superior catalytic performance of Bi_9_O_7.5_S_6_ compared to Bi_2_O_2_S.

To rationalize their different restructuring pathway in acidic media, we propose the following mechanism. The S^2−^ in the [BiS_2_]^−^ layers act as a sacrificial component that is preferentially leached during eCO_2_R, generating metallic Bi domains, while the oxygen‐rich [Bi_2_O_2_]^2+^ slabs are more likely to be preserved and subsequently converted into Bi_2_O_2_CO_3_ by CO_2_‐derived carbonate species. In this way, the interlayer sulfide in Bi_9_O_7.5_S_6_ actively participates in constructing a Bi/Bi_2_O_2_CO_3_ interface, whereas the sulfide in Bi_2_O_2_S does not play such a role. Structural distinction is therefore leading to different structure evolution during eCO_2_R, ultimately resulting in varying catalytic activity and overall performance.

#### 2.3.2 Structural Evolution of Bi_2_MoO_6_ and BiOX (X=Br and Cl) Ns during eCO_2_R

**Figure 6:**
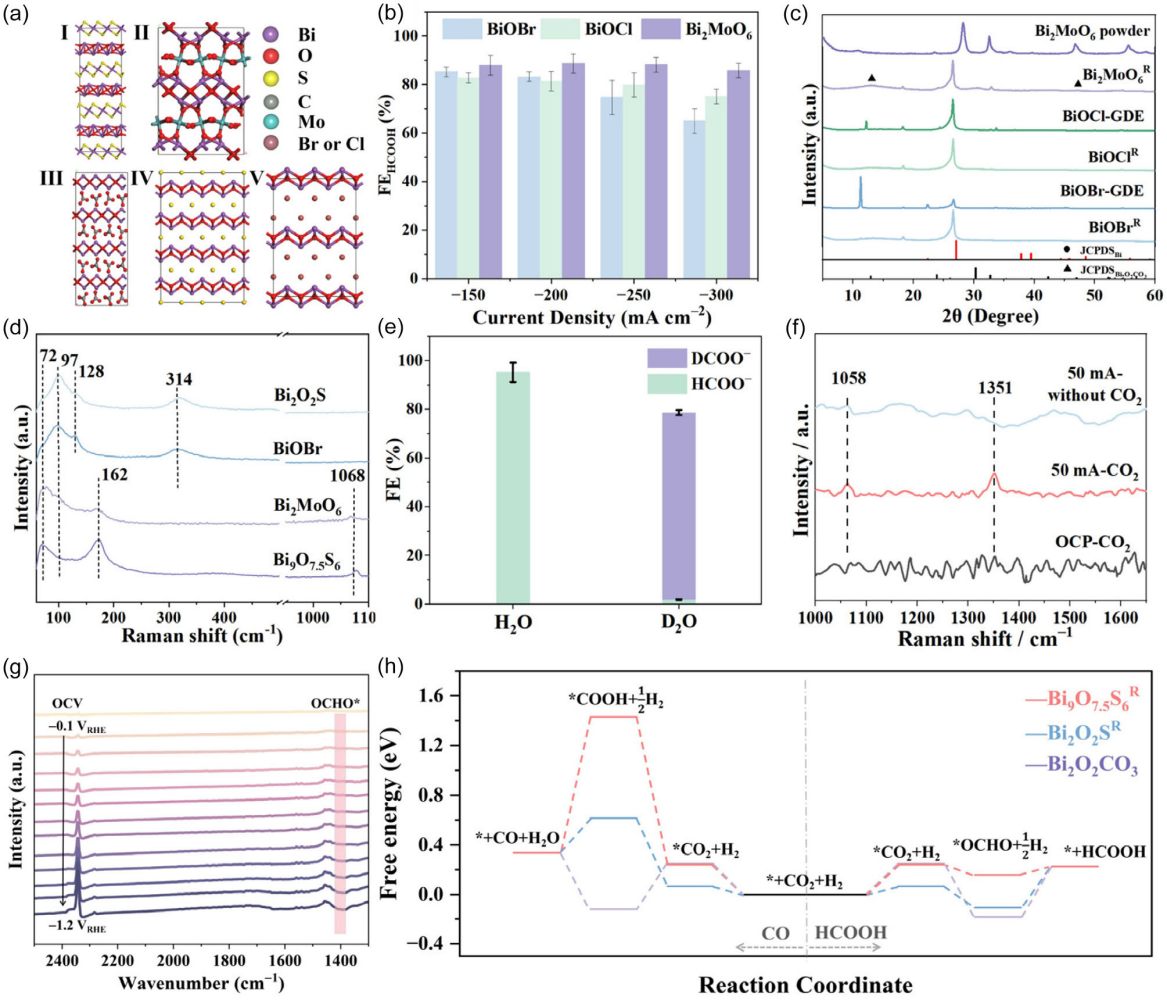
a) The crystal structures of I) Bi_9_O_7.5_S_6_, II) Bi_2_MoO_6_, III) Bi_2_O_2_CO_3_, IV) Bi_2_O_2_S, and V) BiOX (X=Br, Cl). b) eCO_2_R performance of Bi_2_MoO_6_ and BiOX (X=Br, Cl) over a wide current density window in a flow cell. c) XRD patterns of Bi_2_MoO_6_ and BiOX (X=Br, Cl) before and after eCO_2_R. d) Ex situ Raman spectra of different precursors after eCO_2_R at −200 mA cm^−2^. e) FEs of the Bi_9_O_7.5_S_6_ electrode for eCO_2_R to produce HCOOH by using isotope‐labeled D_2_O and isotope nonlabeled H_2_O as electrolyte solvent. f) In situ Raman spectra of intermediates of Bi_9_O_7.5_S_6_ during eCO_2_R under different reaction conditions. g) In situ attenuated total reflection surface‐enhanced infrared absorption spectroscopy (ATR‐SEIRAS) of Bi_9_O_7.5_S_6_ during eCO_2_R under CO_2_ bubbling. h) Gibbs free energy diagram of eCO_2_R to formic acid and CO.

To investigate the influence of Bi/Bi_2_O_2_CO_3_ structure on eCO_2_R performance and clarify the relationship between the restructuring mechanism and its precursor, various Bi‐based materials (Bi_2_MoO_6_, Bi_2_O_2_CO_3_, and BiOX (X=Br and Cl)) were investigated. Bi_2_MoO_6_ has a similar structure to Bi_9_O_7.5_S_6_ with molybdenum (Mo) element in the middle layers, while BiOX (X=Br and Cl) has a similar structure to Bi_2_O_2_S without metal element in the middle layers^[^ ^45^, ^46^ ^]^ (**Figure** **6**a). As shown in Figure 6b, the Bi_2_MoO_6_ catalyst shows an improved eCO_2_R activity at applied current densities compared to the BiOBr and BiOCl catalysts. Bi_2_MoO_6_ can restructure into Bi/Bi_2_O_2_CO_3_ after the reaction (Figure 6c) and yields a FE_HCOOH_ of 90% at a current density of 250 mA cm^−2^ (Figure 6b). Meanwhile, ${\text{CO}}_{3}^{2-}$ ions in the electrolyte spontaneously exchange with ${\text{MoO}}_{4}^{2–}$ in Bi_2_MoO_6_.^[^ ^47^ ^]^ In contrast, BiOCl and BiOBr are restructured into metallic Bi after the reaction (Figure 6c), with their FE_HCOOH_ values of ≈80% and ≈75%, respectively at 250 mA cm^−2^ (Figure 6b). The ex situ Raman results show that peaks appear at 72 and 97 cm^−1^, attributed to Bi—Bi bond in BiOBr, Bi_2_O_2_S, Bi_2_MoO_6_, and Bi_9_O_7.5_S_6_ after the eCO_2_R. In BiOBr and Bi_2_O_2_S, peaks at 128 and 314 cm^−1^ are attributed to surface oxidized β‐Bi_2_O_3_ by environmental oxygen after the eCO_2_R (Figure 6d). In Bi_2_MoO_6_ and Bi_9_O_7.5_S_6_, peaks at 162 and 1068 cm^−1^ gradually appear, attributed to Bi—O bond and C—O bond, respectively. This result reveals that Bi_2_MoO_6_ and Bi_9_O_7.5_S_6_ can be restructured into structures containing excessive Bi—O bonds, attributed to Bi_2_O_2_CO_3_ during the eCO_2_R, consistent with the XRD results in Figure 6c. Therefore, it is speculated that the metal cations contained between the [Bi_2_O_2_]^2+^ layers are less prone to decomposition during eCO_2_R, thereby inhibiting the excessive reduction of [Bi_2_O_2_]^2+^ and retaining more Bi—O bond.

Additionally, Bi/Bi_2_O_2_CO_3_ mixture catalyst was synthesized by mechanical mixing of commercial Bi and Bi_2_O_2_CO_3_ for comparison. Figure S10 and S11, Supporting Information, show the XRD and SEM results before and after eCO_2_R. XRD patterns of the Bi/Bi_2_O_2_CO_3_ mixture show that the emerging peaks of the catalyst correspond well with their standard PDF card (Figure S10a, Supporting Information). After eCO_2_R, according to the XRD results, the catalyst shows the peaks belonging to Bi and Bi_2_O_2_CO_3_ (Figure S10b, Supporting Information). SEM image demonstrates that Bi/Bi_2_O_2_CO_3_ complex consists of irregularly shaped nanosheets with average size ranging between 100 and 500 nm before eCO_2_R (Figure S11a, Supporting Information), while the complex is transformed into nanoflower structure after eCO_2_R (Figure S11b, Supporting Information). As shown in Figure S12, Supporting Information, the complex exhibits FE_HCOOH_ of less than 85% at all applied current densities, while the Bi_9_O_7.5_S_6_^R^ achieves 95% FE_HCOOH_ (Figure 4a) and higher *J*_HCOOH_ (Figure 4c), suggesting the key role of in situ restructured Bi/Bi_2_O_2_CO_3_ for efficient formic acid production.

#### 2.3.3 Reaction Pathway Revealing and Density Functional Theory Calculations

Structural differences affect both the electrochemical active surface area (ECSA) and the intrinsic catalytic activity. To explore the reaction kinetics, we measured the Tafel slopes of the reactions from CO_2_ to formic acid on three catalysts (Figure S13b, Supporting Information), respectively. The Tafel slope of the restructured Bi_9_O_7.5_S_6_ is 48 mV dec^−1^, similar to the value of 58 mV dec^−1^ for Bi/Bi_2_O_2_CO_3_ but smaller than the value of 81 mV dec^−1^ for the restructured Bi_2_O_2_S, indicating the favorable kinetics of CO_2_ conversion on the restructured Bi_9_O_7.5_S_6_. To estimate the ECSA of each electrode, we performed a cyclic voltammetry (CV) test to measure the double‐layer capacitance (*C*_dl_) in a non‐faradic potential window. The *C*_dl_ of the restructured Bi_9_O_7.5_S_6_, Bi_2_O_2_S, and Bi/Bi_2_O_2_CO_3_ is calculated to be 5.32, 0.77, and 3.38 μF cm^−2^, respectively (Figure S14 and S15, Supporting Information). Accordingly, the restructured Bi_9_O_7.5_S_6_ exhibits the largest ESCA. This is one of the reasons that the restructured Bi_9_O_7.5_S_6_ shows better electrocatalytic activities. In summary, all the results indicate that Bi_9_O_7.5_S_6_ outperforms Bi_2_O_2_S in efficient formic acid selectivity because it has more active sites and better kinetics after restructuring.

To further elucidate the mechanism of formic acid formation during eCO_2_R, we explored the kinetic isotope effect (KIE) using its corresponding isotopically labeled reactants D_2_O, which allows for tracing the pathway of hydrogen atoms in the final product formed on the Bi_9_O_7.5_S_6_ catalyst (Figure 6e). The FE of formic acid by using H_2_O as the hydrogen source is higher than that of D_2_O, with a KIE factor greater than 1. This result indicates that proton transfer is involved in the RDS, and the protonation of intermediates occurs at Bi active sites.^[^ ^48^ ^]^ While the specific proton‐transfer pathway remains unidentified, two distinct routes from CO_2_ to HCOOH have been discussed:^[^ ^49^ ^]^ (i) first electron transfer process to form the ${\text{CO}}_{2}^{{*}^{-}}$ radical, followed by proton transfer through the O‐linked pathway to form the *OCHO intermediate, and (ii) proton‐coupled electron transfer (PCET) generation of *COOH intermediates via C‐ligation pathway. The detailed reaction equations are as follows: i) CO_2_R to HCOOH via O‐linked pathway
(1)

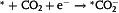



(2)

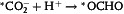



(3)

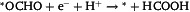

ii) CO_2_R to HCOOH via C‐linked pathway
(4)

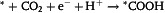



(5)

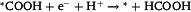




Then, in situ Raman spectra were employed to reveal the intermediate and proton‐transfer pathway under different reaction conditions (Figure S16, Supporting Information). Figure 6f and Figure S17, Supporting Information, display the in situ Raman spectra of Bi_9_O_7.5_S_6_ electrocatalyst with CO_2_‐bubbled 0.05 m H_2_SO_4_ with 0.5 m KCl solution as electrolyte. No peak is observed at open‐circuit potential (OCP) even with CO_2_‐bubbled, while the Raman peak at 1351 cm^−1^ assigned to the O—C—O symmetric vibration in bidentate *OCHO appears and the intensity increases as the applied current increase to 50 mA cm^−2^. Time‐resolved in situ Raman spectra (Figure S17, Supporting Information) further show that this band at 1351 cm^−1^ is the only new feature that grows with reaction time, which strongly correlates with the formation of the formic acid species.^[^ ^50^, ^51^, ^52^ ^]^ No peaks related to *COOH are observed at 1340 and 1450 cm^−1^ under all applied current. Then, in situ ATR‐SEIRAS was performed to investigate the intermediate formation process on the catalyst. As shown in Figure 6g, the peak at 1396 cm^−1^ can be ascribed to the vibration of O—C—O in *OCHO, whose intensity gradually increases as the potential negatively shifts from −0.1 to −1.2 V_RHE_. This evolution suggests that *OCHO is the key intermediate; furthermore, the formation of *HCOOH from the *OCHO is the RDS for converting CO_2_ to formic acid. Combined with isotope labeling experiments, it is determined that the reaction path is an O‐linked path.

As different reaction routes (CO_2_ to HCOOH and CO_2_ to CO) would compete during the eCO_2_R process, the Gibbs free energy profile of various reaction routes was calculated. To verify the active sites in Bi_9_O_7.5_S_6_R, density functional theory calculations were then carried out to investigate the reaction pathways from CO_2_ to HCOOH and made a comparison in Bi_2_O_2_S^R^ and Bi_2_O_2_CO_3_, respectively. The Bi (104) and Bi_2_O_2_CO_3_ (011) surfaces were chosen to represent the Bi_2_O_2_S^R^ and Bi_2_O_2_CO_3_ catalysts, respectively, because XRD and HRTEM of the restructured catalysts consistently identify Bi(104) and Bi_2_O_2_CO_3_ (011) as the predominant facets at the Bi/Bi_2_O_2_CO_3_ interface of Bi_9_O_7.5_S_6_^R^. The Bi cluster deposited on the Bi_2_O_2_CO_3_ (011) surfaces represents the Bi/Bi_2_O_2_CO_3_ interface in the Bi_9_O_7.5_S_6_^R^ catalyst. Herein, the Bi (104) surface and Bi_2_O_2_CO_3_ (011) surface are intended to simulate the experimentally observed Bi/Bi_2_O_2_CO_3_ structure and to capture the local coordination environment of the active sites. These models nevertheless provide only a simplified description of the real catalyst surface. The surfaces and adsorption structures are generated on the LASPAI website.^[^ ^53^, ^54^, ^55^, ^56^ ^]^ The adsorption configurations of reaction intermediates on different catalysts were exhibited in Figure S18, Supporting Information. And the energy barrier of RDS for various routes is compared to unveil the preference of products. As shown in Figure 6h, the reaction energies of the RDSs on the Bi_9_O_7.5_S_6_^R^, Bi_2_O_2_S^R^, and Bi_2_O_2_CO_3_ surfaces are 0.07 eV, 0.33 eV, and 0.41 eV, respectively. The free energy change from *OCHO to HCOOH on Bi_9_O_7.5_S_6_^R^ is the most energetically favorable, compared with that on Bi_2_O_2_S^R^ and Bi_2_O_2_CO_3_, indicating that the Bi/Bi_2_O_2_CO_3_ interface is the most possible active site in the Bi_9_O_7.5_S_6_^R^ catalysts to produce HCOOH. On the other hand, the reaction from CO_2_ to CO on the Bi_9_O_7.5_S_6_^R^ catalyst is not feasible as the reaction energy from CO_2_ + H_2_ to COOH + ½ H_2_ is over 1 eV, supporting the role of Bi_9_O_7.5_S_6_^R^ to selectively produce HCOOH and suppress the formation of CO.

## 3 Conclusion

In this study, structurally distinct Bi‐based oxysulfur compounds were employed to achieve different reconstructed electrocatalysts, allowing us to explore the structure‐activity relationship in acidic electrolytes for eCO_2_R through the combined use of theoretical calculations and catalytic experiments. Quasi‐in situ X‐ray diffraction and in situ Raman spectra reveal that the presence of metal elements within the interlayers of the precursors dictates their restructuring into a Bi/Bi_2_O_2_CO_3_ composite structure. Metal elements situated between two [Bi_2_O_2_]^2+^ layers can resist decomposition and prevent the over‐reduction of catalysts. Consequently, Bi_9_O_7.5_S_6_ is in situ restructured into Bi_2_O_2_CO_3_ with metallic Bi, preserving the active interface between Bi and Bi_2_O_2_CO_3_, which is favorable for formic acid production. Furthermore, theoretical calculations demonstrate that the active interface of Bi_9_O_7.5_S_6_R favored the conversion of *OCHO to HCOOH, which is the rate‐determining step of CO_2_ electrocatalytic reduction to formic acid. As a result, Bi_9_O_7.5_S_6_ demonstrates superior activity and selectivity for converting CO_2_ to formic acid. This work presents a strategy for controlling catalyst restructuring through compositional modification, thereby enhancing both the activity and stability of the catalysts. This strategy could be further employed in other catalytic systems to enhance sustainable chemical and fuel production.

## Supporting Information

Supporting Information is available from the Wiley Online Library or from the author.

## Conflict of Interest

The authors declare no conflict of interest.

## Supporting information

Supplementary Material

## Data Availability

The data supporting this article have been included as part of the Supplementary Information.
