# Supplementary material for: Restructuring‐Regulated Bismuth Catalyst Promotes Electrochemical CO2 Reduction to Formic Acid in Acidic Electrolyte
Source: Small Sci. 2026 Jan 19;6(1):e202500565. doi: 10.1002/smsc.202500565 (PMC12822384; doi:10.1002/smsc.202500565)
Supplement: Supplementary file 1 — Supplementary Material [file SMSC-6-e202500565-s001.pdf]

# Supporting Information

## Restructuring-regulated Bismuth Catalyst Promotes Electrochemical $CO_2$ Reduction to Formic Acid in Acidic Electrolyte

Ganwen Chen[a,b#], Chun Liu[b#], Jie Chen[c#], Yukun Xiao[b,d], Yumin Da[b], Meng Wang[b,e], Chenrui Ji[b], Jie He[a], Rongjie Xu[a], Lei Fan[b*], Zhangliu Tian[b,f*] and Wei Chen[a,b,g*]

[a] Joint School of National University of Singapore and Tianjin University, International Campus of Tianjin University, Binhai New City, Fuzhou 350207, PR China

[b] Department of Chemistry, National University of Singapore, 3 Science Drive 3, 117543, Singapore

[c] State Key Laboratory of Metal Organic Chemistry, Shanghai Institute of Organic Chemistry, Chinese Academy of Sciences, Shanghai, 200032, China

[d] Northwest Institute for Non-ferrous Metal Research, Xi'an 710016, People's Republic of China.

[e] Advanced Institute for Materials Research (WPI-AIMR), Tohoku University, Sendai, 980-8577 Japan

[f] CAS Key Laboratory of Materials for Energy Conversion, Shanghai Institute of Ceramics, Chinese Academy of Sciences, Shanghai, 200050, PR China

[g] Department of Physics, National University of Singapore, 2 Science Drive 3, 117542, Singapore

[*] Corresponding Author E-mail: leifan@nus.edu.sg; tianzhangliu@mail.sic.ac.cn; phycw@nus.edu.sg (Wei CHEN)

**Table of Contents**

Experimental Section ........................................................................................................ 4

Materials Synthesis........................................................................................................ 4

Physical Characterizations.............................................................................................. 4

Electrochemical measurement......................................................................................... 5

Supplemental Figures ........................................................................................................ 6

Figure S1. STEM-EDS elemental mapping of the (a) $Bi_9O_{7.5}S_6$ and (b) $Bi_2O_2S$. ................ 7

Figure S2. Linear sweep voltammetry (LSV) curves of Bi, $Bi_2O_2CO_3$ and $Bi_9O_{7.5}S_6$ under $CO_2$ and Ar atmospheres in acid electrolyte.......................................................................... 8

Figure S3. $eCO_2R$ performance of $Bi_9O_{7.5}S_6$ in different electrolytes: (a) 0.05M $H_2SO_4$ + 0.1M KCl and (b) 0.05 M $H_2SO_4$ + 1M KCl. (c) Corresponding voltages of $Bi_9O_{7.5}S_6$ at different current densities and pH value of the electrolyte in 0.05 M $H_2SO_4$ + 0.5M KCl. (d) Corresponding FE and potentials at different pH values and –100 mA $cm^{-2}$ current density. ........................................................................................................................... 9

Figure S4. *Quasi-in-situ* XRD patterns for structural evolution of $Bi_9O_{7.5}S_6$ and $Bi_2O_2S$ NSs during $eCO_2R$. .............................................................................................................. 10

Figure S5. (a) SEM image, (b) TEM image of $Bi_9O_{7.5}S_6$ after stability test....................... 11

Figure S6. (a) TEM image, (b) STEM-EDS elemental mapping, (c) Ex-situ XRD patterns of $Bi_9O_{7.5}S_6$ after stability test. ............................................**Error! Bookmark not defined.**

Figure S7. (a) Ex-situ XRD patterns of $Bi_2O_2S$, (b) SEM image, (c) TEM image of $Bi_2O_2S$ after $eCO_2R$. .......................................................................**Error! Bookmark not defined.**

Figure S8. XPS spectra of (a) Bi 4*f* and S 2*p*, (b) O 1*s* and (c) S 2*s* of the $Bi_9O_{7.5}S_6$ catalyst after $eCO_2R$. .......................................................................**Error! Bookmark not defined.**

Figure S9. XPS spectra of (a) Bi 4*f* and S 2*p*, (b) O 1*s* and (c) S 2*s* of the $Bi_2O_2S$ catalyst after $eCO_2R$. ........................................................................................................... 15

Figure S10. XRD patterns of mechanical mixing Bi and $Bi_2O_2CO_3$ (a) before (b) after $eCO_2R$..................................................................................**Error! Bookmark not defined.**6

Figure S11. SEM images of mechanical mixing Bi and $Bi_2O_2CO_3$ (a) before (b) after $eCO_2R$.............................................................................................................................. 17

Figure S12. $eCO_2R$ performance of mechanical mixing Bi and $Bi_2O_2CO_3$ under 0.05 M $H_2SO_4$ with 0.5 M KCl as electrolyte in flow cell.............................................................. 18

Figure S13. (a) Linear sweep voltammetry (LSV) curves of $Bi_9O_{7.5}S_6$, $Bi/Bi_2O_2CO_3$ and $Bi_2O_2S$ under $CO_2$ and Ar atmospheres in acid electrolyte. (b) Tafel slopes of the restructured $Bi_9O_{7.5}S_6$, $Bi/Bi_2O_2CO_3$ and $Bi_2O_2S$ NSs........................................................ 19

Figure S14. (a) The restructured $Bi_2O_2S$ and (c) $Bi_9O_{7.5}S_6$ CV curves with different scan rates. (b) The restructured $Bi_2O_2S$ and (d) $Bi_9O_{7.5}S_6$ $C_{dl}$ by dividing the double layer charging current differences with the scan rates. ................................................................ 20

Figure S15. (a) The restructured $Bi/Bi_2O_2CO_3$ CV curves with different scan rates. (b) The restructured $Bi/Bi_2O_2CO_3$ $C_{dl}$ by dividing the double layer charging current differences with the scan rates. ........................................................................................................... 21

Figure S16. Photos and schematic of the silicon prism and *in-situ* ATR-SEIRAS setup. (a) Silicon prisms coated with a thin Au film by chemical deposition. (b) Silicon prism after deposition of the catalyst on the Au-coated surface. (c) *In-situ* ATR-SEIRAS measurement device used in this work. (d) Schematic illustration of the *in-situ* ATR-SEIRAS setup. .... 22

Figure S17. Time dependent *in-situ* Raman spectra of intermediates of $Bi_9O_{7.5}S_6$ during $eCO_2R$. ........................................................................................................................... 23

Figure S18. The optimized structures with adsorbed intermediates on (a-e) $Bi_9O_{7.5}S_6^R$, (f-j) $Bi_2O_2S^R$ and (k-o) $Bi_2O_2CO_3$. The intermediates are: (a, f, k) $CO_2$, (b, g, l) *OCHO, (c, h, m) HCOOH, (d, i, n) *COOH, (e, j, o) CO.................................................................................. 24

Supplemental Tables

Table S1. Some reported Bi-based electrocatalysts for $eCO_2R$ to formate in acid electrolyte. ............................................................................................................................ 25

## Experimental Section

**Synthesis of $Bi_9O_{7.5}S_6$ Ns.** 0.01 mol $Bi(NO_3)_3{\cdot}5H_2O$ (4.85 g)、0.01 mol Thiourea (0.79 g)、KOH (5g) and LiOH (10g) were added to a mixed solvent containing 5 mL ultrapure water and 5 mL ethanol followed by manually stirring until mixed evenly. Afterward, the contents were transferred into a 50 ml Teflon-lined stainless-steel autoclave and heated at 180°C for 72 h. After naturally cooling to room temperature, the dark grey precipitate was washed with $H_2O$ and ethanol and then dried in oven at 60°C.

**Synthesis of $Bi_2O_2S$ Ns.** 0.48 g CTAB was first added to 50 mL ultrapure water followed by 30mins magnetic stirring. Then $Bi(NO_3)_3{\cdot}5H_2O$ (0.2328 g) was subsequently added with continuous stirring for 45 mins. Afterward, Thiourea (0.0183 g) was added to the solvent with continuous stirring for 15 mins. Lastly, LiOH (2.68 g) was added into the solvent followed by ~45mins continuous stirring. The mixed solution was placed into a 100 ml Teflon-lined stainless-steel autoclave and hydrothermally treated at 200°C for 90 mins. After naturally cooling to room temperature, the brick red precipitate was alternately washed with DI water and ethanol and then dried in oven at 60°C.

**Synthesis of $Bi_2O_2CO_3$ Ns.** 0.68 mmol $Bi(NO_3)_3{\cdot}5H_2O$ (0.332 g) was dissolved in 50 mL ultrapure water followed by ~30 mins ultrasonic treatment. Then $Na_2CO_3$ (73 mg) was added into the solvent with continuous stirring for 1h. Afterward, the contents were transferred into a 100 ml Teflon-lined stainless-steel autoclave and heated at 150°C for 12 h. After naturally cooling to room temperature, the dark grey precipitate was washed with $H_2O$ and ethanol and then dried in oven at 60°C.

**Synthesis of $Bi/Bi_2O_2CO_3$ Ns** 1 mmol Bi (0.209 g) and 1 mmol $Bi_2O_2CO_3$ (0.510 g) weremixed in 5 mL ethanol and ball-milled for 3 h forward and 3 h reverse. Then the mixture was washed with DI water and ethanol and then dried in oven at 60°C.

**Characterizations.** The samples were tested by X-ray diffractometer (XRD, Cu Kα, λ = 1.5405 Å, D2 PHASER, Bruker), field emission SEM (FE-SEM, JEOL JSM6700F), XPS (ESCALAB 250Xi) with Al K*a* X-ray as the excitation source, Raman spectra (Renishaw InVia Qontor confocal Raman Microscope) with 532 nm laser excitation and high-resolution

transmission electron microscopy (HRTEM, FEI Tecnai G2F30) equipped with an X-ray energy dispersive spectrometer (EDS).

***Quasi-in-situ* XRD measurements.** Quasi-in-situ XRD experiments were performed using flow cells based on original electrocatalytic tests. After the corresponding activation time and different conditions, the electrolyte is immediately removed and XRD testing is performed.

***In-situ* Raman measurements.** In-situ Raman spectra experiments were performed using a top-plate cell photic apparatus linked to an electrocatalytic workstation. The experimental setup employed the same three-electrode configuration (working electrode (WE), reference electrode (RE), and counter electrode (CE)) and electrolyte solution as previously utilized in the electrocatalytic experiments. Before initiating Raman spectra measurements, $CO_2$ gas was infused into the electrolyte to ensure saturation. The in-situ Raman spectra were collected using a Renishaw InVia Qontor confocal Raman Microscope, which was equipped with a 532 nm laser excitation source.

***In-situ* ATR-SEIRAS measurement.** Prior to the spectrum test, the silicon (Si) prism specifically designed for *in-situ* ATR-SEIRAS measurements was cleaned and coated with a thin layer of Au film using a chemical deposition method described in the literature, as shown in Fig. S17a. After the deposition, the obtained Au film on the silicon prism was rinsed with deionized water and dried with air. The catalyst ink consisted of 9 mg of catalysts, 80 μL of 5 wt% Nafion, and 820 μL of isopropyl alcohol. The working electrode was prepared by depositing a total of 100 μL of the ink mixture onto the surface of the silicon prism, as shown in Fig. S16b. The working electrode was placed in a two-compartment with a three-electrode electrochemical cell. In order to avoid pollution from the counter electrode, the working electrode (catalyst on Si prism) and the reference electrode (Ag/AgCl in saturated KCl solution) were separated from a graphite rod as the counter electrode by an anion exchange membrane. The only stirring effect inside the cell was from various bubbled gases. The cell was integrated into a Fourier Transform Infrared Shimadzu spectrometer (IR Tracer 100) for the ATR-SEIRAS measurements (Fig. S16c-d). The electrochemical measurements were performed using a CHI 760E instrument. The experiments were carried out in a 0.05 M

$H_2SO_4$ with 0.5 M KCl solution as electrolyte at room temperature. Prior to the measurements, liquid nitrogen was added into the spectrometer. To ensure $CO_2$ saturation, a constant flow of $CO_2$ gas was purged into the electrolyte for 1 hour before in situ ATR-SEIRAS measurements, and the $CO_2$ flow was maintained throughout the electrochemical experiments. The spectra were presented in transmission mode, with negative peaks indicating an increase in the signal and positive peaks indicating a decrease in the signal. After in situ ATR-SEIRAS measurements for each catalyst sample, the silicon prism was washed with an aqua regia solution to remove Au and the catalyst from its surface.

**Electrochemical measurements.** The $CO_2$R performances of the various samples were tested by using a three-electrode flow cell system immersed in a $CO_2$-saturated aqueous solution containing 0.05 M $H_2SO_4$ and 0.5 M KCl. Electrochemical measurements were performed on a CHI 760E electrochemical workstation (CH Instruments, Shanghai, China). And all the e$CO_2$R performances were measured after the stable LSV scanning. All potentials were calculated to the RHE with the following equation: $E_{RHE} = E_{Ag/AgCl} + 0.197 + 0.059 \times pH$, and then compensated with iR. For the working electrode (WE), approximately 1 mg of the catalyst was deposited on a gas diffusion layer covering a 1 $cm^2$ area. An Ag/AgCl electrode and a Ni foam served as the reference electrode (RE) and the counter electrode (CE), respectively. During the measurement, $CO_2$ was pumped into the cathode chamber with a constant flow rate (20 $mL•min^{-1}$). The gas products were detected by using an in-situ connected gas chromatograph instrument (GC, Shimadzu 2014C). Liquid products were identified by a 1H-NMR spectrum (Bruker AVNEO 500 MHz system). The Faradaic efficiency (FE) during $CO_2$R is calculated by the equation, $FE = Q_i/Q_t = (N_i \times n \times F)/Q_t$, where Qi represents the charge amount associated with the reduction of the product, Qt is the total charge consumed, Ni is the product molar amount, n is the number of electrons transferred (which is 2 for formate, $H_2$ and CO), and F is the Faradaic constant (96,485 $C•mol^{-1}$).

## Supplemental Figures

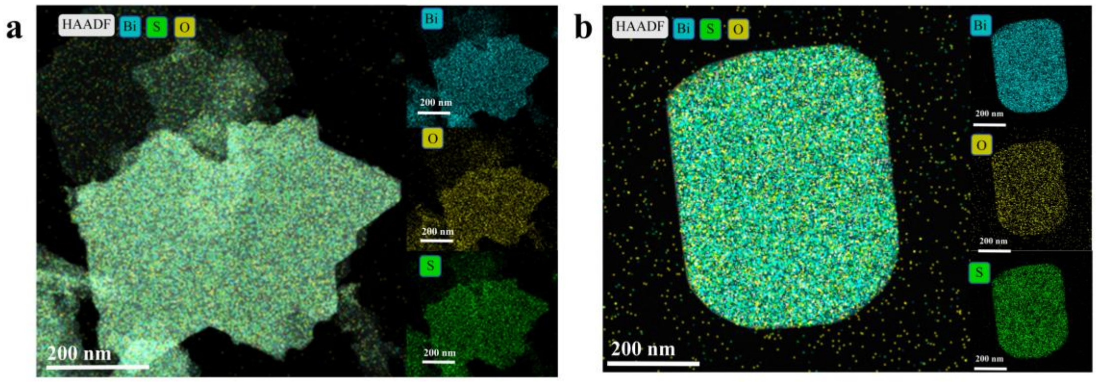


**Figure S1** STEM-EDS elemental mapping of the (a) $Bi_9O_{7.5}S_6$ and (b) $Bi_2O_2S$.

**Supplementary Note 1** The energy dispersive X-ray spectroscopy (EDX) elemental mapping images of $Bi_9O_{7.5}S_6$ and $Bi_2O_2S$ confirm a uniform distribution of O, S, and Bi elements throughout the whole nanosheet.

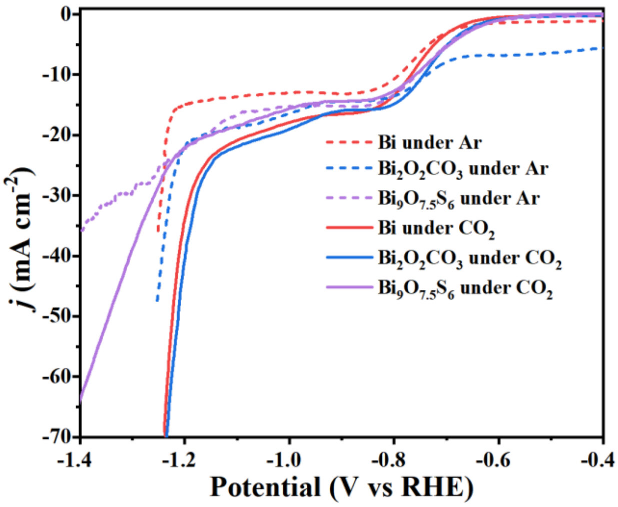


**Figure S2** Linear sweep voltammetry (LSV) curves of Bi, $Bi_2O_2CO_3$ and $Bi_9O_{7.5}S_6$ under $CO_2$ and Ar atmospheres in acid electrolyte.

**Supplementary Note 2** Linear sweep voltammetry (LSV) curves of the pristine Bi, $Bi_2O_2CO_3$ and $Bi_9O_{7.5}S_6$ were conducted to evaluate their $eCO_2R$ performance over a potential range of –0.4 to –1.4 $V_{RHE}$, where a three-electrode flow cell reactor was employed with 0.05 M $H_2SO_4$ as the anode electrolyte and 0.05 M $H_2SO_4$ with 0.5 M KCl as the cathode electrolyte, respectively.

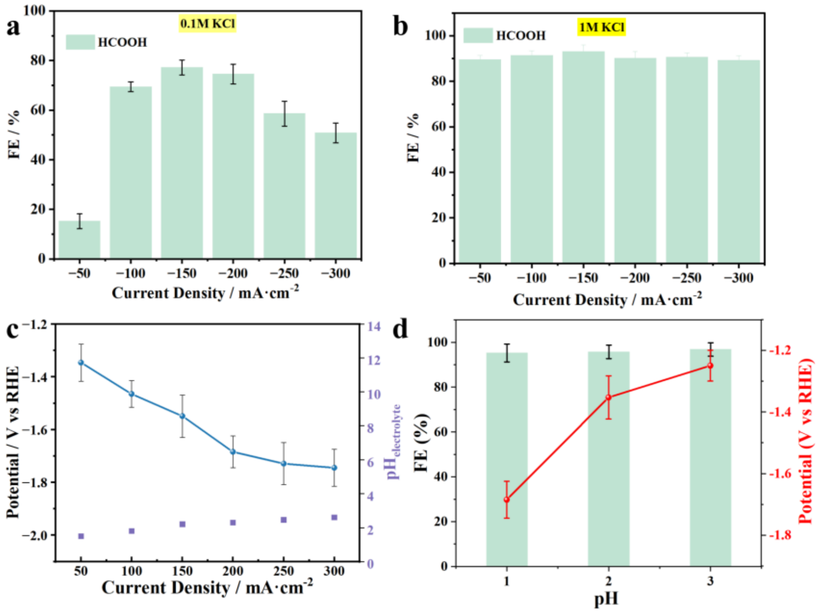


**Figure S3** $eCO_2R$ performance of $Bi_9O_{7.5}S_6$ in different electrolytes: (a) 0.05M $H_2SO_4$ + 0.1M KCl and (b) 0.05 M $H_2SO_4$ + 1M KCl. (c) Corresponding voltages of $Bi_9O_{7.5}S_6$ at different current densities and pH value of the electrolyte in 0.05 M $H_2SO_4$ + 0.5M KCl. (d) Corresponding FE and potentials at different pH values and –100 mA cm$^{-2}$ current density.

**Supplementary Note 3** (a-b) This experiment aims to investigate the influence of $K^+$ concentration in the electrolyte on the electrocatalytic performance of the $Bi_9O_{7.5}S_6$ catalyst. (c-d) Other $eCO_2R$ performance, such as the calculated $J_{HCOOH}$ and the pH value during the reaction are also evaluated.

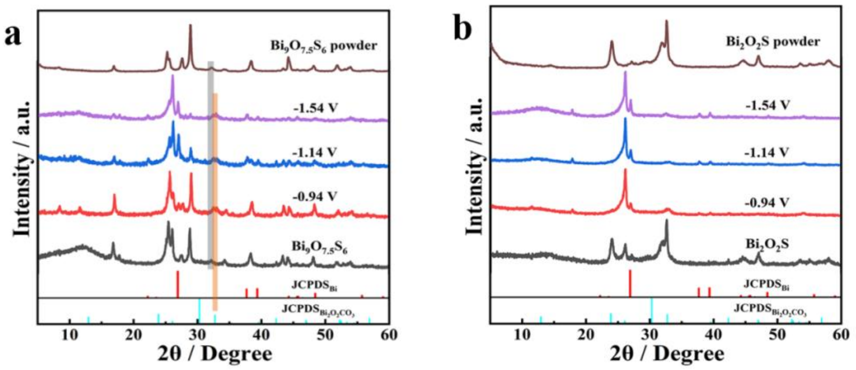


**Figure S4** *Quasi-in-situ* XRD patterns for structural evolution of $Bi_9O_{7.5}S_6$ and $Bi_2O_2S$ NSs during $eCO_2R$.

**Supplementary Note 4** *Quasi-in-situ* XRD was carried out during $eCO_2R$ for $Bi_9O_{7.5}S_6$ and $Bi_2O_2S$ NSs at different potentials. This result aligns with the conclusion of **Figure 4d and 4g**. The gray and orange bar from all the XRD patterns highlight the peaks of $Bi_9O_{7.5}S_6$ and $Bi_2O_2CO_3$, respectively.

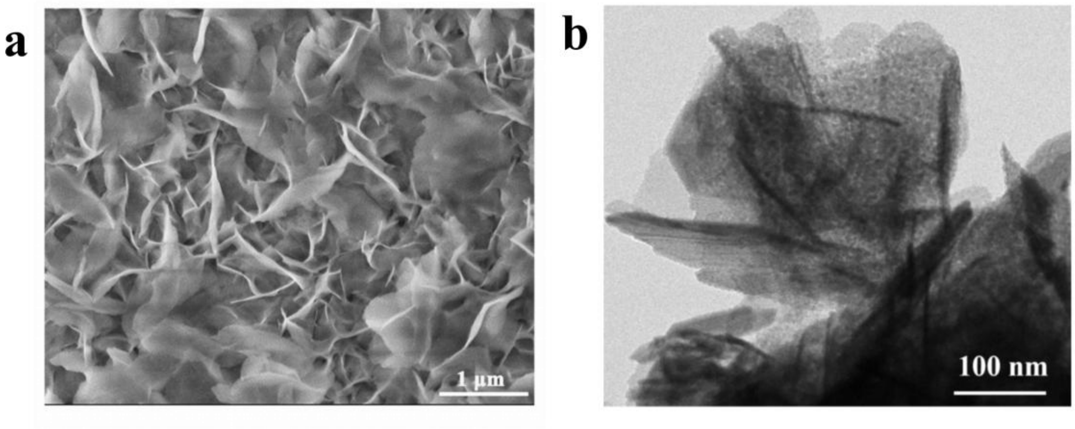


**Figure S5** (a) SEM image, (b) TEM image of $Bi_9O_{7.5}S_6$ after stability test.

**Supplementary Note 5** The XRD pattern of $Bi_9O_{7.5}S_6$ after stability test consistent with the results after performance test.

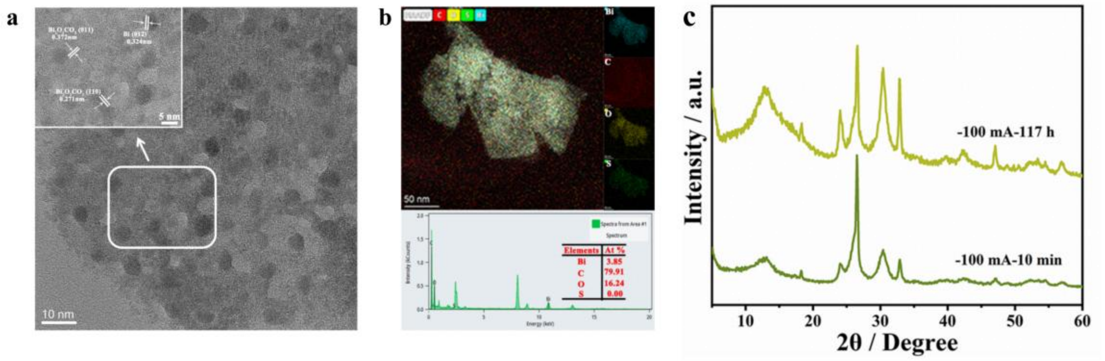


**Figure S6** (a) TEM image, (b) STEM-EDS elemental mapping, (c) Ex-situ XRD patterns of $Bi_9O_{7.5}S_6$ after stability test.

**Supplementary Note 6** The XRD pattern of $Bi_9O_{7.5}S_6$ after stability test consistent with the results after performance test.

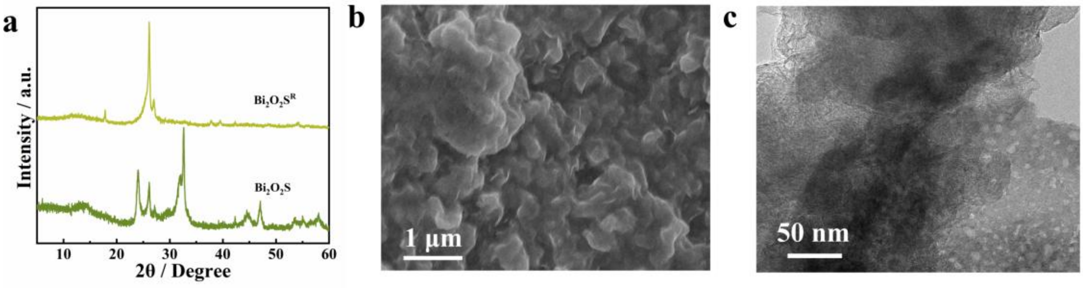


**Figure S7** (a) Ex-situ XRD patterns of $Bi_2O_2S$, (b) SEM image, (c) TEM image of $Bi_2O_2S$ after $eCO_2R$.

**Supplementary Note 7** The result is consistent with the results of *Quasi*-*in*-*situ* XRD patterns of the $Bi_2O_2S$ electrode at 50 mA $cm^{-2}$ for different times and applied potentials.

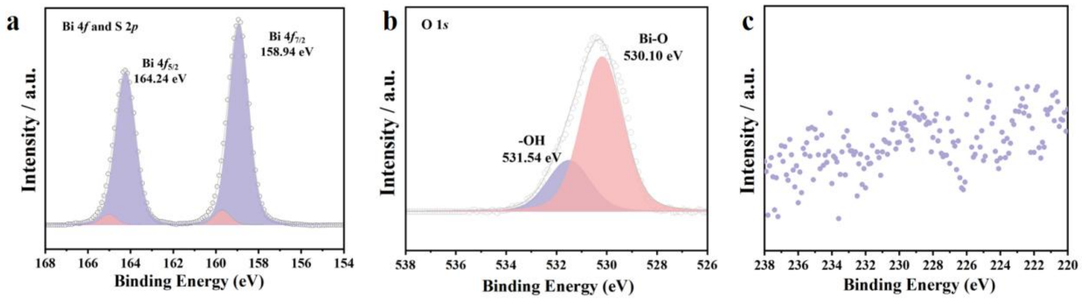


**Figure S8** XPS spectra of (a) Bi 4*f* and S 2*p*, (b) O 1*s* and (c) S 2*s* of the $Bi_9O_{7.5}S_6$ catalyst after $eCO_2R$.

**Supplementary Note 8** Dominant peaks in Bi 4*f* spectra (**Figure S8a**) can be deconvoluted into four subpeaks at 158.94, 159.72, 164.24, 165.02 eV, respectively. The O 1s XPS spectrum of the restructured $Bi_9O_{7.5}S_6$ (**Figure S8b**) can be split into three deconvolution peaks at approximately 530.1 and 531.54 eV, which belong to Bi-O and Bi-OH, respectively. The 4*f* orbital range of Bi coincides with the 2*p* orbital range of S. However, the peaks of S 2*p* cannot be recognized in this range. At the same time, the characteristic peak of S 2*s* (**Figure S8c**) cannot be measured either.

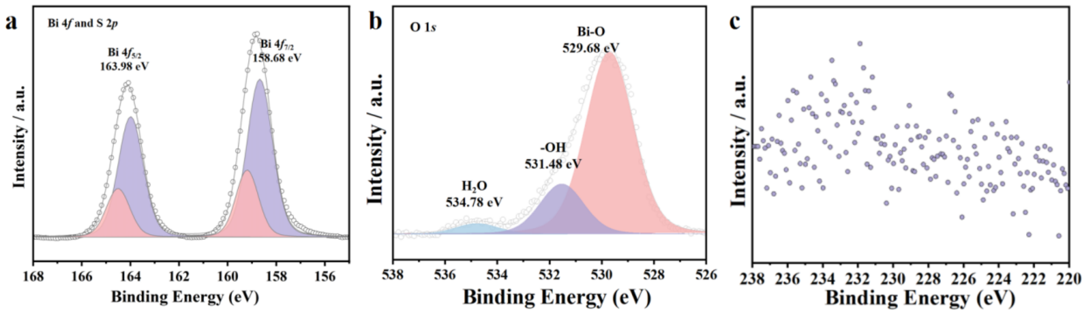


**Figure S9** XPS spectra of (a) Bi 4*f* and S 2*p*, (b) O 1*s* and (c) S 2*s* of the $Bi_2O_2S$ catalyst after $eCO_2R$.

**Supplementary Note 9** Dominant peaks in Bi 4*f* spectra (**Figure S9a**) can be deconvoluted into four subpeaks at 158.68, 159.18, 163.98, 164.48 eV, respectively. The O 1s XPS spectrum of the restructured $Bi_9O_{7.5}S_6$ (**Figure S9b**) can be split into three deconvolution peaks at approximately 529.68, 531.48 eV and 534.78, which belong to Bi-O, Bi-OH, and surface-adsorbed water, respectively. The 4*f* orbital range of Bi coincides with the 2*p* orbital range of S. However, the peaks of S 2*p* cannot be recognized in this range. At the same time, the characteristic peak of S 2*s* (**Figure S9c**) cannot be measured either.

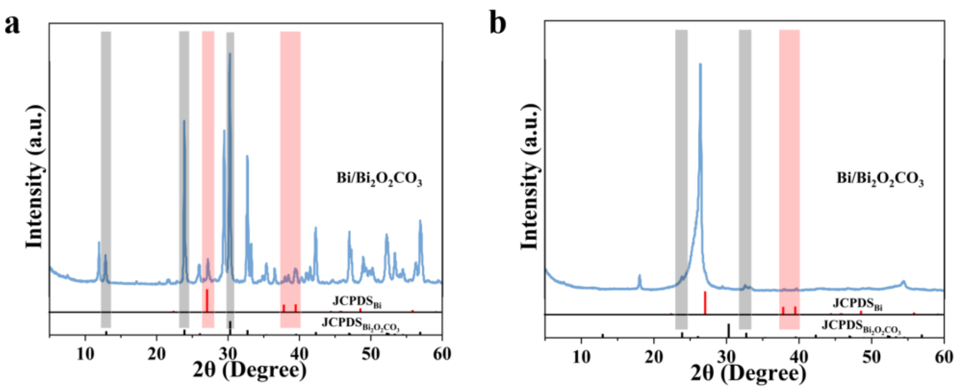


**Figure S10** XRD patterns of mechanical mixing Bi and $Bi_2O_2CO_3$ (a) before (b) after $eCO_2R$.

**Supplementary Note 10** *Ex-situ* XRD was carried out before and after $eCO_2R$ for mechanical mixing Bi and $Bi_2O_2CO_3$. The gray and red bar point out the peaks of $Bi_2O_2CO_3$ and Bi, respectively.

a b

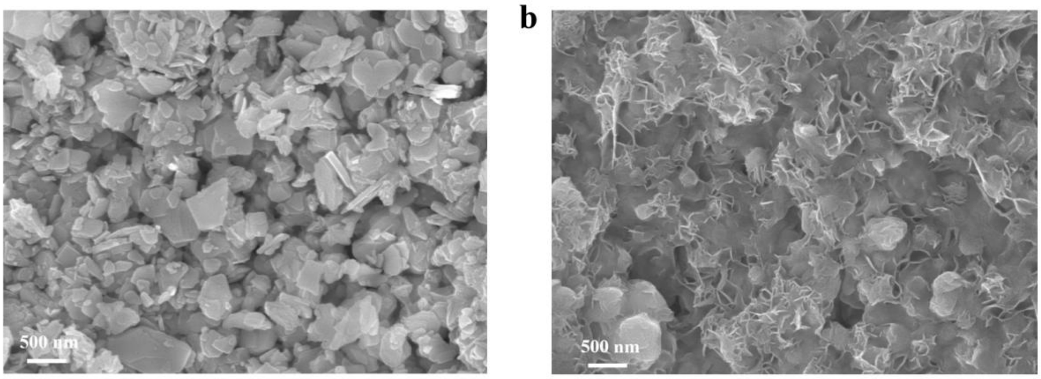


**Figure S11** SEM images of mechanical mixing Bi and $Bi_2O_2CO_3$ (a) before (b) after $eCO_2R$.

**Supplementary Note 11** SEM image shows that the $Bi/Bi_2O_2CO_3$ complex consists of irregularly shaped nanosheets, with sizes ranging from 100 to 500 nm before $eCO_2R$. After $eCO_2R$, the complex is transformed into nanoflower structure, with 500 nm scale bar.

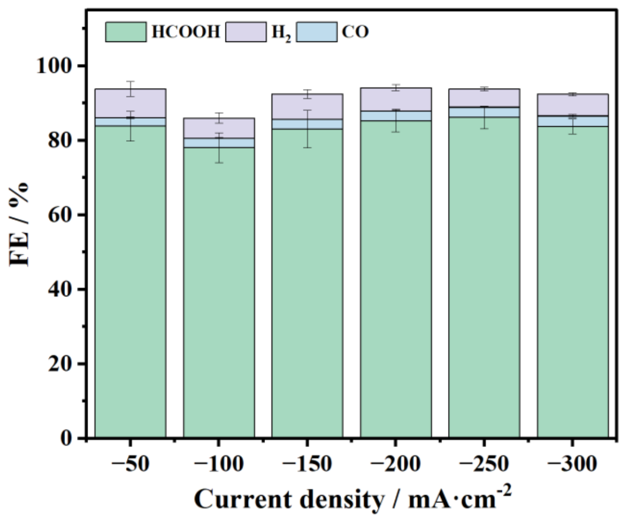


**Figure S12** $eCO_2R$ performance of mechanical mixing Bi and $Bi_2O_2CO_3$ under 0.05 M $H_2SO_4$ with 0.5 M KCl as electrolyte in flow cell.

**Supplementary Note 12** The $eCO_2R$ performance was tested in the same test environment as $Bi_9O_{7.5}S_6$ and error bars were also added.

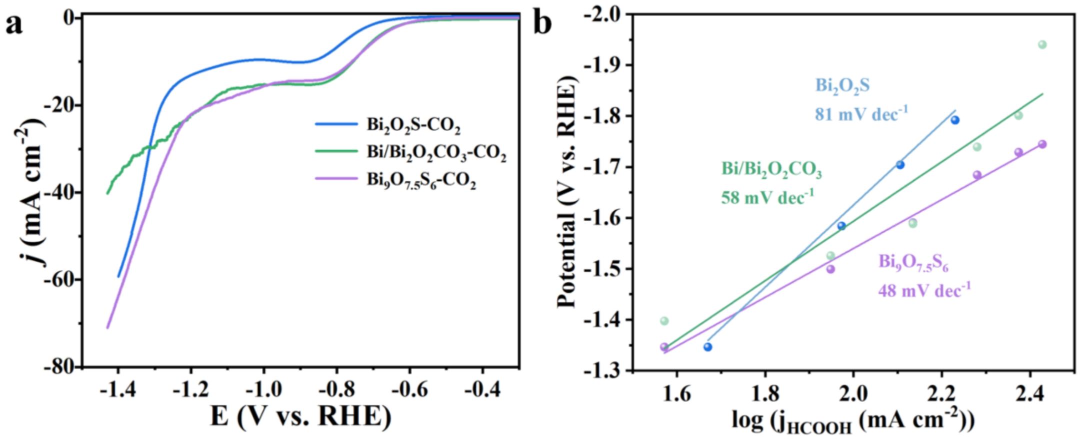


**Figure S13** (a) Linear sweep voltammetry (LSV) curves of $Bi_9O_{7.5}S_6$, $Bi/Bi_2O_2CO_3$ and $Bi_2O_2S$ under $CO_2$ and Ar atmospheres in acid electrolyte. (b) Tafel slopes of the restructured $Bi_9O_{7.5}S_6$, $Bi/Bi_2O_2CO_3$ and $Bi_2O_2S$ NSs.

**Supplementary Note 13** To explore the reaction kinetics, we measure the Tafel slope by using the Linear sweep voltammetry (LSV) curves and logarithm of formic acid partial current density against the applied potentials to evaluate the reaction kinetics of $eCO_2R$.

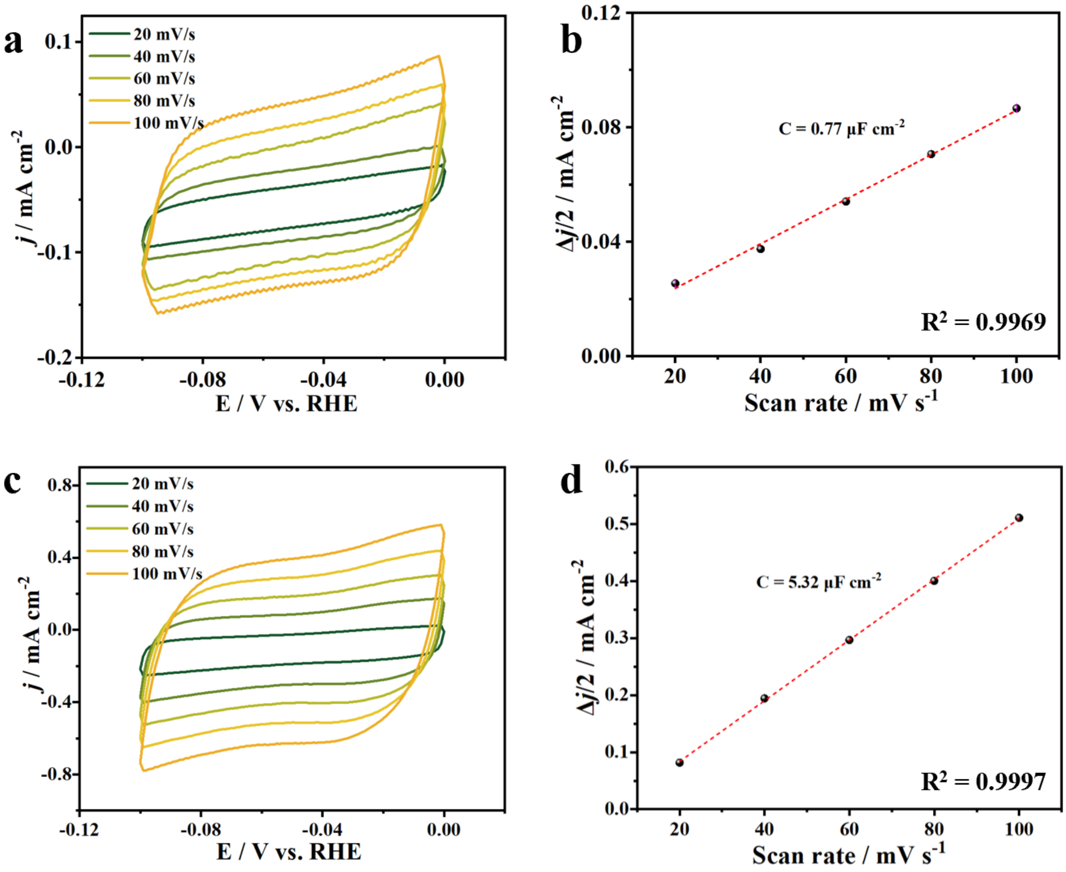


**Figure S14** The CV curves of restructured (a) $Bi_2O_2S$ and (c) $Bi_9O_{7.5}S_6$ with different scan rates. The $C_{dl}$ of restructured (b) $Bi_2O_2S$ and (d) $Bi_9O_{7.5}S_6$, obtained from the slopes of the linear fits of the the double layer charging current differences versus the scan rates. The coefficients of determination ($R^2$) for the linear fits are as follows: $R^2$=0.9969 for $Bi_2O_2S^R$ and $R^2$=0.9997 for $Bi_9O_{7.5}S_6^R$.

**Supplementary Note 14** The electrochemically active surface area (ECSA) was estimated from the double-layer capacitance ($C_{dl}$) using cyclic voltammetry (CV) in a non-Faradaic potential region. The capacitive current density difference was determined as:

$$\Delta j = j_{\text{anodic}} - j_{\text{cathodic}}$$

where $j_{\text{anodic}}$ and $j_{\text{cathodic}}$ are the current densities obtained from the anodic and cathodic sweeps at the same potential, respectively. To isolate the capacitive component, we plotted ($\Delta$j/2) as a function of the scan rate (v). Because the capacitive current is proportional to v, the linear fit of ($\Delta$j/2) vs. v yields a slope equal to the $C_{dl}$.

Finally, the ECSA was estimated using:

$$\text{ECSA} = C_{dl}/C_s$$

where $C_s$ is the specific capacitance of a smooth planar surface in the same electrolyte.

The electrochemical double electric layer capacitances ($C_{dl}$) of the restructured $Bi_9O_{7.5}S_6$ and $Bi_2O_2S$ were calculated to be 5.32, 0.77 and 3.38 μF $cm^{-2}$.

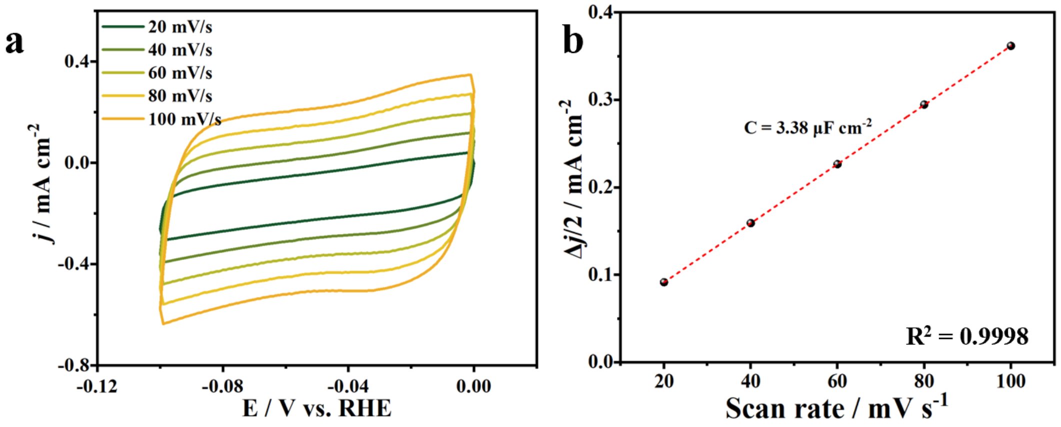


**Figure S15** (a) The CV curves of restructured Bi/$Bi_2O_2CO_3$ with different scan rates. (b) The $C_{dl}$ of restructured Bi/$Bi_2O_2CO_3$, obtained from the slopes of the linear fits of the the double layer charging current differences versus the scan rates. The coefficient of determination ($R^2$) for the linear fit is $R^2$=0.9998 for Bi/$Bi_2O_2CO_3^R$.

**Supplementary Note 15** The electrochemical double electric layer capacitances ($C_{dl}$) of the restructured Bi/$Bi_2O_2CO_3$ is calculated to be 3.38 μF $cm^{-2}$.

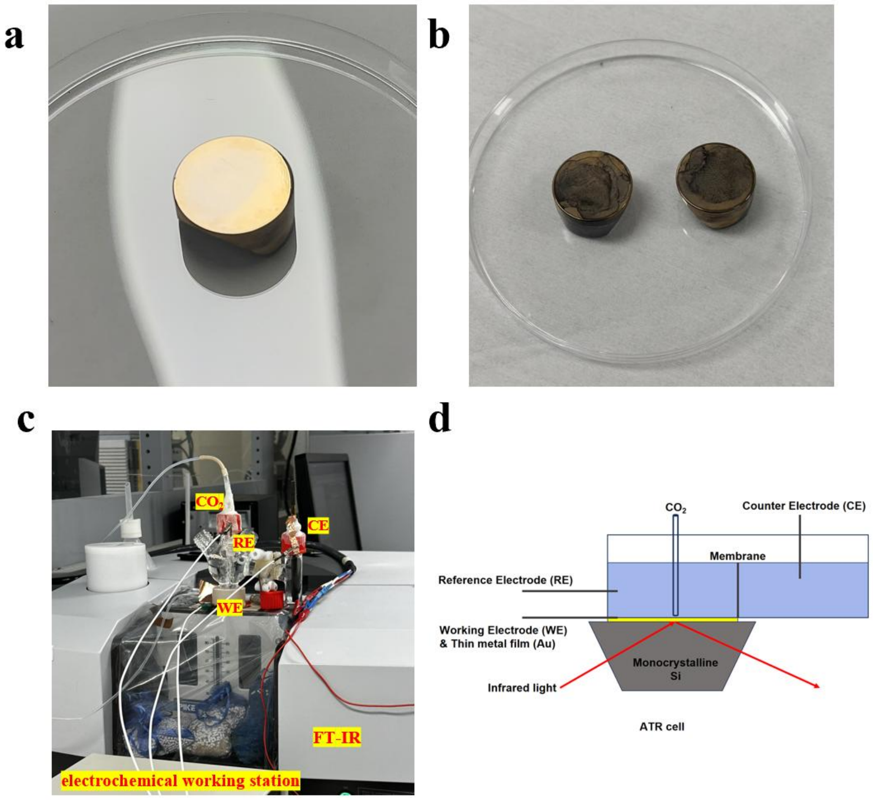


**Figure S16.** Photos and schematic of the silicon prism and *in-situ* ATR-SEIRAS setup. (a) Silicon prisms coated with a thin Au film by chemical deposition. (b) Silicon prism after deposition of the catalyst on the Au-coated surface. (c) *In-situ* ATR-SEIRAS measurement device used in this work. (d) Schematic illustration of the *in-situ* ATR-SEIRAS setup.

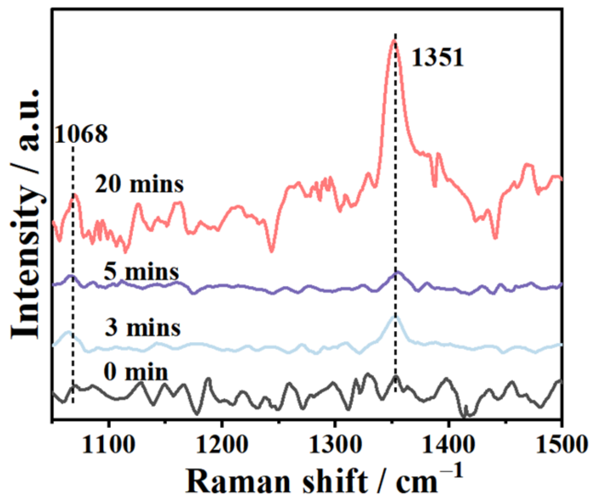


**Figure S17** Time dependent *in-situ* Raman spectra of intermediates of $Bi_9O_{7.5}S_6$ during $eCO_2R$.

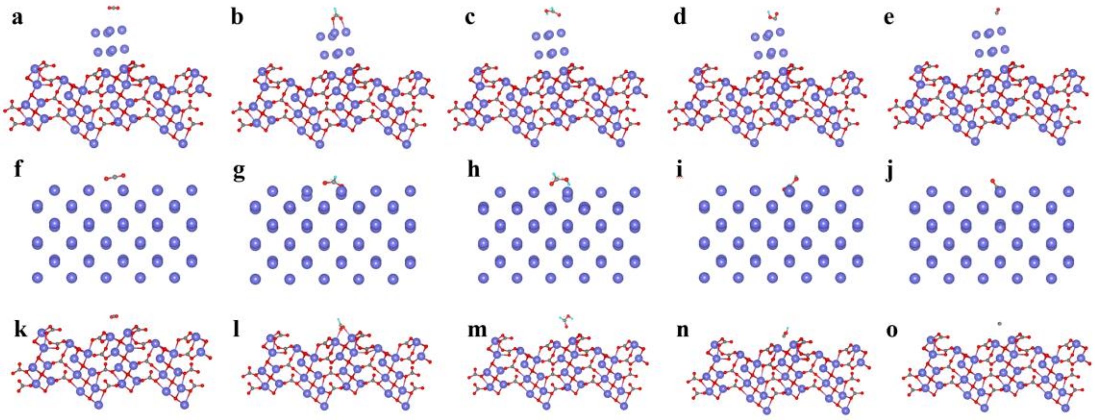


**Figure S18**. The optimized structures with adsorbed intermediates on (a-e) $Bi_9O_{7.5}S_6^R$, (f-j) $Bi_2O_2S^R$ and (k-o) $Bi_2O_2CO_3$. The intermediates are: (a, f, k) $CO_2$, (b, g, l) *OCHO, (c, h, m) HCOOH, (d, i, n) *COOH, (e, j, o) CO.

# Supplemental Tables

**Table S1**: Some reported Bi-based electrocatalysts for e$CO_2$R to formate in acid electrolyte.

| Catalysts | Cell | Electrolyte | pH | Potential (V vs RHE) | FE (%) | $J_{HCOOH}$ (mA $cm^{-2}$) | Stability (mA $cm^2$,h) | R (mmol $h^{-1}cm^{-2}$) | CEE (%) | Ref. |
|---|---|---|---|---|---|---|---|---|---|---|
| **Jagged Bi nanosheets** | **Flow** | **0.5 M $K_2SO4$ & 0.05 M $H_2SO_4$** | **1.2** | **–1.2** | **90.7** | **≈ 110** | **——** | **3.35** | **≈ 55** | **35** |
| **3D-Bi-TDPE-PPSU** | **H** | **1M $Na_2SO_4$** | **2.7** | **–1.26** | **85.2** | **63.6** | **40, 25** | **1.18** | **44.1** | **36** |
| **Bimetallic Cu-Bi** | **Flow** | **1 M KCl & $H_2SO_4$** | **2** | **–1.0** | **92** | **98** | **100, 8** | **1.86** | **51.9** | **37** |
| **Bi nanosheets** | **Flow** | **3M KCl & 0.05 M $H_2SO_4$** | **0.5** | **–1.28** | **92.2** | **237.1** | **≈120, 8** | **4.42** | **52.4** | **38** |
| | | **0.5M KCl & 0.05 M $H_2SO_4$** | **0.83** | **–1.23** | **≈ 20** | **≈ 12** | **——** | **0.223** | **11.6** | |
| **$Cs_3Bi_2Br_9$** | **H** | **HBr-CsBr aqueous** | **2.5** | **–0.95** | **92** | **4.1** | **4.3, 20** | **≈ 0.149** | **0.6** | **39** |
| **NU-1000-Sn** | **Flow** | **0.005 M $H_2SO_4$ & 3 M KCl** | **1.67** | **–1.8** | **95** | **247** | **260, 15** | **4.6** | **44.8** | **40** |
| **PGDY@I-BOCR** | **H** | **0.5 M $K_2SO_4$** | **3.5** | **–1.4** | **94.8** | **60** | **60, 240** | **1.84** | **51.5** | **23** |
| **$Bi_9O_{7.5}S_6$-derived** | **Flow** | **0.5M KCl & 0.05 M $H_2SO_4$** | **0.9** | **–1.68** | **95.3** | **190** | **100, 117** | **5.02** | **48.9** | **This work** |
